# Sex differences in *Drosophila* intestinal metabolism contribute to sexually dimorphic infection outcome and alter gut pathogen virulence

**DOI:** 10.1073/pnas.2514992123

**Published:** 2026-06-24

**Authors:** Marko Rubinić, Yi Yu, Aranzazu Arias-Rojas, Kaisy A. Martinez, Wioletta Klimek, Dagmar Frahm, Volker Brinkmann, Nicole Paczia, Kathirvel Alagesan, David Duneau, Igor Iatsenko

**Affiliations:** ^a^ Research group Genetics of host-microbe interactions, Max Planck Institute for Infection Biology, Berlin 10117, Germany; ^b^ Faculty of Life Sciences, Humboldt-Universität zu Berlin, Berlin 10099, Germany; ^c^ Microscopy Core Facility, Max Planck Institute for Infection Biology, Berlin 10117, Germany; ^d^ Core Facility for Metabolomics and Small Molecule Mass Spectrometry, Max Planck Institute for Terrestrial Microbiology, Marburg 35043, Germany; ^e^ Max Planck Unit for the Science of Pathogens, Berlin 10117, Germany; ^f^ Centre for Cardiovascular Science, Queen’s Medical Research Institute, University of Edinburgh, Edinburgh EH16 4TJ, United Kingdom; ^g^ Center for Ecology, Evolution and Environmental Changes and Global Change and Sustainability Institute, Faculty of Sciences, University of Lisbon, Lisbon 1749-016, Portugal

**Keywords:** *Drosophila melanogaster*, sexual dimorphism, intestinal infection, Pseudomonas, reactive oxygen species

## Abstract

Sexual dimorphism in infection outcomes is widespread, yet its underlying mechanisms remain incompletely understood. Utilizing **Pseudomonas* entomophila* intestinal infection in *Drosophila,* we demonstrate that sex differences in intestinal redox processes contribute to female-biased susceptibility to infection. Female inability to overcome pathogen-induced oxidative stress results in defecation blockage, pathogen persistence, and host death. Male flies exhibit increased carbohydrate metabolism and pentose phosphate pathway activity—a key antioxidant defense system. This allows males to withstand oxidative stress-induced defecation blockage and clear the pathogen from the intestine, resulting in survival. Notably, we uncovered that Duox-dependent processes contribute to pathology independently of total ROS levels. In parallel, *P. entomophila* showed increased expression of several virulence factors, including RNA-binding protein Hfq, in the female gut, contributing to female-biased virulence of *P. entomophila*. Thus, the effect of the gut metabolic environment on host defenses and pathogen virulence determines the sex differences in intestinal infection outcomes.

## Significance

Sex differences in susceptibility to infections are a common yet understudied phenomenon across animals. Here, we showed that female *Drosophila* are more susceptible to intestinal infection due to their inability to cope with specific infection-induced stress, resulting in defecation blockage, pathogen persistence, and host death. In contrast, higher expression of nicotinamide adenine dinucleotide phosphate (NADPH)-producing enzymes in male flies helps them to mitigate oxidative stress, clear the pathogen, and survive the infection. Furthermore, we uncovered that the pathogen shows heightened virulence in the female gut due to the augmented abundance of several virulence factors. These findings shed light on the critical interplay among host metabolism, intestinal defenses, and pathogen virulence in shaping sex-specific differences in infection outcomes.

The sex is a determinant parameter of phenotypic and physiological heterogeneity of the organism (1, 2). Sex differences in immune defenses, response to infections, and anti-infective treatments have been often reported (3–5). However, the mechanisms underlying these differences remain mostly unresolved. Such lack of knowledge is not surprising, as it stems from the prevalent practice of utilizing only one sex in research studies. Understanding the complex interplay between sex and immunity has been further complicated by the fact that the dimorphism is often pathogen-specific, can be affected by the environment (6, 7), and depends not only on the host but also on the sex-specific pathogen response (8–10).

Sexual dimorphism in response to gut infections remains particularly understudied, despite the accumulating evidence that certain gastrointestinal pathogens infect the host in sex-biased manner (6). The intricate interplay among immune responses, microbiota, behavior, environment, and genetic factors ultimately shapes the sex bias observed in the intestinal response to infections (11, 12). Given the complexity of these interactions, it is not surprising that a comprehensive mechanistic understanding of sexual dimorphism in the outcomes of intestinal infections remains elusive.

*Drosophila melanogaster* is a powerful model to study host–microbe interactions due to a wide array of genetic tools that allow for the fine manipulation of cells and tissues both spatially and temporally (13, 14). Considering the increasing appreciation of sexually dimorphic physiology of fruit flies, they gain attention as a model to understand sexual dimorphism in immunity and infection outcome (2, 3, 7, 15–18). The evolutionary conservation of key defense mechanisms in fruit flies (13, 14) allows for extrapolation of findings to other organisms, highlighting the broader relevance of insights gained from *Drosophila* studies on immune sex dimorphism.

Fruit flies rely on several defense mechanisms against intestinal pathogens (13, 14). The peritrophic matrix that shields epithelial cells from pathogens (19–21) and the acidic region in the middle midgut that eliminates the ingested microbes via acid secretion (22–24) represent physical barriers. The production of antimicrobial peptides (AMPs) and additional immune effectors in specific regions of the gut represents an inducible arm of defense (25–27). While the Immune deficiency pathway (Imd) is a key regulator of antimicrobial response in the midgut of flies, subset of AMPs, like Drosomycin-like 2 (Drsl2) and Drosomycin-like 3 (Drsl3) are controlled by the JAK-STAT pathway (25). The Toll pathway functions specifically in the foregut and hindgut with less pronounced role in intestinal immunity (13) but with a key role in sexual dimorphism to systemic infections (7). Reactive oxygen species (ROS) are also rapidly produced in the gut by the Duox enzyme in the response to pathogen-secreted uracil (28). While these ROS might be microbicidal against some microbes (29), there is accumulating evidence that Duox-produced ROS have signaling role in promoting gut peristalsis and clearance of the pathogens via defecation (30–33). A number of additional signaling pathways are activated upon intestinal cell damage to initiate stem cell proliferation, tissue repair (25, 34, 35), and resilience mechanisms (36). While the described mechanisms were identified in females, we do not know whether and how they differ in males.

**Pseudomonas* entomophila* originally isolated from fruit flies (37) is one of the few microbes that can establish lethal infection in the *Drosophila* gut (38). *P. entomophila* can block intestinal defenses by producing toxins and proteases that degrade AMPs and compromise the integrity of peritrophic matrix (39, 40). During female *Drosophila* response to *P. entomophila*, excessive ROS accumulation results in oxidative stress, leading to translation blockage, nonreversible gut damage, and death of the majority of female flies (26, 37, 39, 41). *P. entomophila* pathogenesis has not yet been described in male flies. Additionally, differences between males and females in intestinal metabolic processes (42, 43) might distinctly affect pathogen virulence, thus contributing to sexual dimorphism in infection susceptibility.

We studied in-depth the sex differences in *Drosophila* susceptibility to intestinal *P. entomophila* infection. We showed that male bias in basal gut NADPH-producing enzyme levels contributes to the antioxidant response to gut infection, favoring regular intestinal transit, bacterial clearance, and overall better survival than female flies. Overall, our findings reveal a mechanism by which carbohydrate metabolism shapes sexual dimorphism in susceptibility to infection by affecting ROS’s effects on the host and the pathogen.

## Results

### *Drosophila* Susceptibility to Gut Infection Is Sexually Dimorphic.

**Fig. 1.**
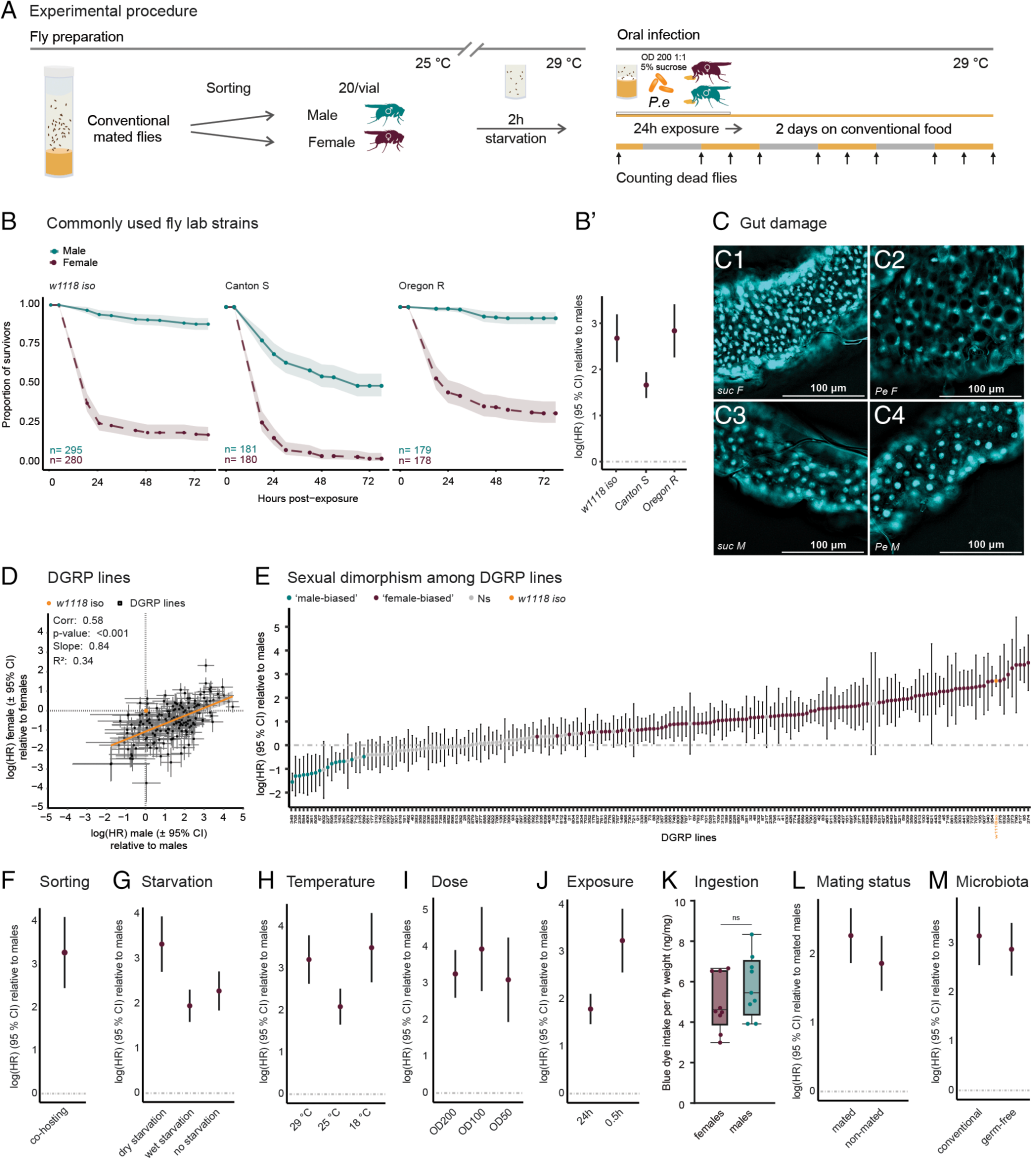
*Drosophila* susceptibility to gut infection is sexually dimorphic. (*A*) Graphical overview of experimental procedure to investigate sex differences in survival to intestinal infection. (*B* and *B’*) Survival curves with 95% CI (shaded area) and hazard ratios with 95% CI of commonly used lab strains upon exposure to *P. entomophila*. (*C*) Representative images of Hoechst staining of the gut 16 h after treatment; panels *C1* and *C3* show sucrose-treated female and male guts, respectively, while *C2* and *C4* show the corresponding infected guts. (*D*) A scatter plot representing correlation in the variation of hazard ratios of female and male flies (each relative to the appropriate sex of the *w^1118^* iso strain) within the same DGRP line. (*E*) Variability in sexual dimorphism in susceptibility to *P. entomophila* infection across 183 DGRP lines (DGRP lines where female flies were more susceptible compared to male flies are highlighted in plum, those where male flies were more susceptible compared to female flies are highlighted in teal. The reference *w^1118^* iso strain is in orange despite the female’s bias). See *SI Appendix*, Fig. S1*C* for a larger version. (*F*–*J*) Hazard ratios with 95% CI of *w^1118^* iso upon exposure to *P. entomophila* under different protocol conditions; (*F*) cohosting, (*G*) dry starvation, wet starvation, and no starvation, (*H*) 29 °C, 25 °C, and 18 °C, (*I*) OD200, OD100, and OD50, (*J*) 24 h and 0.5 h. (*K*) Intake of blue dye mixed with *P. entomophila* during 0.5 h of exposure of female and male *w^1118^* iso flies. Each dot represents a pool of five flies taken from different infection vials, and the experiment was independently performed on three different days. (*L*) Hazard ratios with 95% CI of mated and nonmated *w^1118^* iso females upon exposure to *P. entomophila* compared to mated *w^1118^* iso males. (*M*) Hazard ratios with 95% CI of conventional and germ-free *w^1118^* iso females upon exposure to *P. entomophila* compared to appropriate male flies. Throughout the panels, error bars represent 95% CI. Nonoverlapping CIs between treatments or genotypes indicate statistically significant differences (*P* < 0.05). Similarly, when a 95% CI does not overlap with the dashed reference lines, it indicates a significant difference from the reference value (generally female).

To study to which extent *Drosophila* is sexually dimorphic in susceptibility to intestinal infection, we infected male and female flies of the same genetic background by feeding them with a mix of *P. entomophila* pathogen suspension and sucrose using a previously established protocol (26) (Fig. 1*A*). We observed that the vast majority of male flies of three commonly used *Drosophila* lab strains; *w^1118^* iso, Canton S, and Oregon R, survived *P. entomophila* infection, while 70 to 80% of female flies were killed (Fig. 1 *B* and *B′*). Hoechst staining of the midgut epithelium in *P. entomophila*–infected *Drosophila* males reveals preserved epithelial organization. Unlike females, males do not display extensive cell loss, nuclear disorganization, or large regions of epithelial disruption, indicating a relative resistance to infection-induced gut pathology (Fig. 1*C*). To further demonstrate the generality of sexual dimorphism in susceptibility to gut infection among genetically distinct individuals, we tested survival postexposure to *P. entomophila* in environment-controlled conditions of 183 *Drosophila* melanogaster Genetic Reference Panel (DGRP) lines (44) allowing to test the proportion of genotypes with a given proportion of dimorphism. As anticipated from a previous study (41), we observed variation among DGRP lines in susceptibility to *P. entomophila* in both sexes (Fig. 1*D* and *SI Appendix*, Fig. S1 *A* and *B*). Correlation between hazard ratios of both sexes shows a high ratio of positive linear relationships between the sex and survival outcomes following an infection (Fig. 1*E* and *SI Appendix*, Fig. S1*C*). Since we tested male and female flies of each DGRP line in parallel, we could calculate the sexual dimorphism in survival for each line (Fig. 1*E*). Among the 120 lines that showed statistically significant sexually dimorphic susceptibility, 87.5% (105/120) exhibited phenotype observed with lab strains—*P. entomophila* being more lethal to female files.

Next, we tested how stable sexual dimorphism in susceptibility to *P. entomophila* infection is under different protocol conditions. Sexual dimorphism was equally pronounced when male and female flies were infected and cohosted in the same vial and when they were infected and separated in different vials (Fig. 1*F* and *SI Appendix*, Fig. S2*A*). Thus, sex differences in survival are not due to potential variability between vials in infectivity or due to the effect of isolation. Since typical oral infection protocol includes a 2 h starvation step in an empty vial (dry starvation), which might negatively affect females more than males, we tested alternative starvation strategies. We still observed increased susceptibility of female flies to infection following wet starvation (vials with 1% agar) or without starvation (Fig. 1*G* and *SI Appendix*, Fig. S2*B*). We also found that dimorphism was still striking at different temperatures, although stronger at 29 °C and 18 °C (Fig. 1*H* and *SI Appendix*, Fig. S2*C*). Furthermore, differences in gut infection can occur due to differences in feeding behavior that were previously reported between sexes (45, 46). To test this, we compared sexual dimorphism using a lower dose of *P. entomophila* and exposed flies to *P. entomophila* for 0.5 h (referred to as 0.5 h protocol) to ensure that the potential difference in continuous ingestion does not explain the sexual dimorphism. Differences between males and females in survival were present when different doses of *P. entomophila* were used (Fig. 1*I* and *SI Appendix*, Fig. S2*D*) and in the 0.5 h protocol (Fig. 1*J* and *SI Appendix*, Fig. S2*E*). Finally, we confirmed that initial pathogen ingestion in the 0.5 h protocol does not statistically differ between sexes (Fig. 1*K*). This suggests that the difference in susceptibility is not due to a difference in initial pathogen intake. Given a known trade-off between reproduction and immunity (47), we tested whether females might be more susceptible to infection due to higher investment in reproduction at the expense of immunity. As expected, virgin females, compared to mated ones, survived *P. entomophila* infection better (*SI Appendix*, Fig. S2 *F* and *F′*). However, they were still more susceptible than males (Fig. 1*L* and *SI Appendix*, Fig. S2*F*). Thus, the increased susceptibility of females to infection is not due to the immunosuppressive effect of reproduction. Susceptibility to gut infection can also be affected by the microbiota composition (23, 48). However, germ-free flies showed sex differences comparable to conventional flies in susceptibility to infection (Fig. 1*M* and *SI Appendix*, Fig. S2*G*), excluding the possibility that sexual dimorphism is determined by microbiota.

### Previously Described Defense Pathways Are Not the Leading Cause of Sex Differences in Susceptibility.

**Fig. 2.**
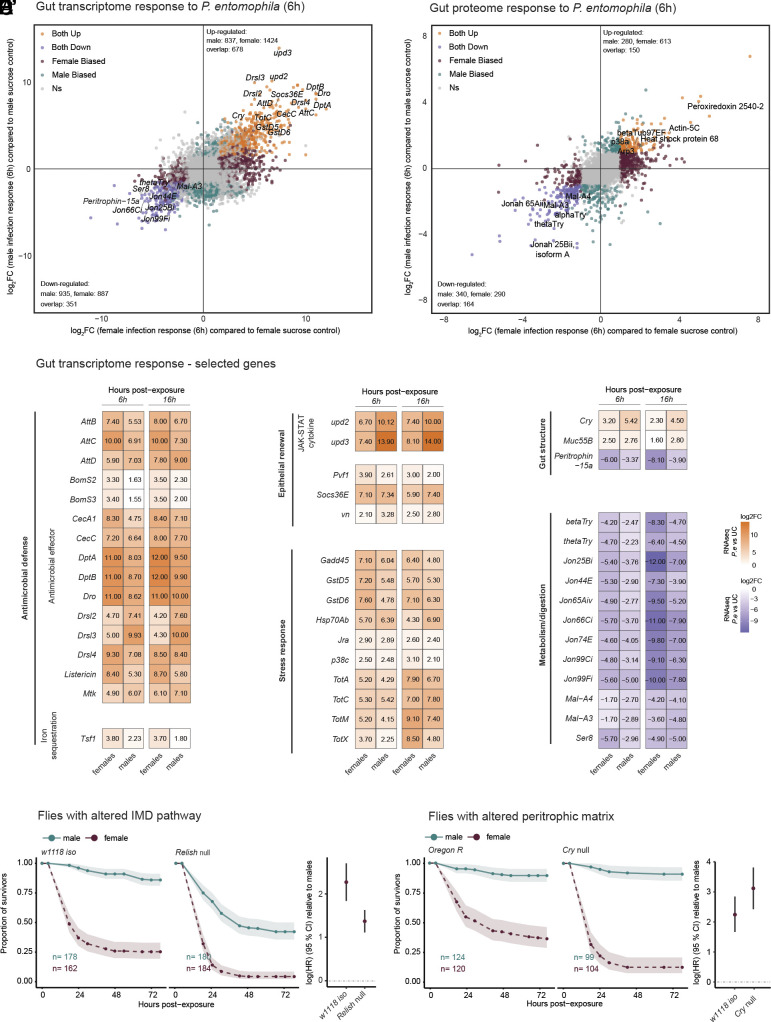
Previously described defense pathways are not the leading cause of sex differences in susceptibility. (*A* and *B*) Scatter plots representing log_2_FC of (*A*) gene expression or (*B*) protein abundance in female (x) vs. male (y) guts 6 h postexposure to *P. entomophila* (infected guts compared to sucrose-fed (control) guts of matching sex). Significance cut-offs: (*A*) padj < 0.1, |log_2_FC| > 1.5, and (*B*) padj < 0.05, |log_2_FC| > 1. (*A*) (N = 3 independent samples, each with 30 pooled guts). (*B*) (N = 5 independent samples, each with 30 pooled guts). (*C*) Heatmaps showing differences (log_2_FC, infected guts compared to control guts) in the expression of selected genes of each sex at 6 h and 16 h postexposure to *P. entomophila*. (*D* and *D’*) Survival curves with 95% CI (shaded area) and hazard ratios with 95% CI of *w^1118^* iso (control) and *Relish^E20^* loss-of-function mutant upon exposure to *P. entomophila*. (*E* and *E’*) Survival curves with 95% CI (shaded area) and hazard ratios with 95% CI of *Oregon R* (control) and *Cry* loss-of-function mutant upon exposure to *P. entomophila*.

Since intestinal immune defenses have not yet been characterized in male flies, we investigated the transcriptional response to *P. entomophila* infection of male alongside of female flies. We performed bulk RNA-seq of dissected guts of infected male or female *w^1118^* iso flies at 6 h and 16 h time points after exposure to *P. entomophila*. Differential expression analysis between pathogen-exposed and nonexposed controls was performed within each sex (Fig. 2*A* and *SI Appendix*, Fig. S3*A* and Table S1). Our initial analysis aimed to identify commonalities in the transcriptional response and later we also describe sexually biased processes. We observed a lower number of significantly upregulated genes in males (6 h: 837 in males vs. 1,424 in females, 16 h: 1,414 males vs. females 1,564). However, 81% (678 out of 837) of all genes induced in males at 6 h postinfection was also upregulated in females (see *SI Appendix*, Table S1 for a list of overlapping and unique genes). Although the overlap was lower for 16 h 64% (913 out of 1,564), these results show overlay in the transcriptional response of males and females to *P. entomophila* infection. Specifically, we found that males and females share upregulated genes involved in antimicrobial defense (AMPs, Tsf1), stress response (Turandots, Hsps, Gstds), stem cell activation, and epithelial renewal (EGFR and JAK-STAT pathways), gut structure (Cry, peritrophin) (Fig. 2 *A* and *C* and *SI Appendix*, Fig. S3*A* and Table S1). Genes encoding digestive enzymes were repressed after infection in both sexes. These results are consistent with a previous study that analyzed the female response to *P. entomophila* infection (26) and suggest that sexual dimorphism in survival is unlikely primarily due to sex differences in these processes.

Since *P. entomophila* causes translation blockage in female flies (26), we additionally undertook a proteomics approach under the same conditions as RNAseq to look for potential differences at the proteome level. Similarly to RNAseq, we observed a higher number of upregulated proteins in female flies (6 h: 278 in males vs. 611 in females, 16 h: 359 males vs. females 911). Consistent with transcriptomics, we detected substantial overlap in the proteome response of males and females to *P. entomophila* infection [6 h: 53.6% (149/278), 16 h: 79.7% (296/359), see *SI Appendix*, Table S2 for a list of proteins]. Among proteins that were upregulated in both sexes, we detected stress response proteins (heat shock proteins, p38a, peroxiredoxin 2540), proteins with a role in the cytoskeleton and epithelial renewal (betaTub56D, betaTub97EF, Tubulin beta-3 chain, Actin-related protein 3, Actin-5C) (Fig. 2*B* and *SI Appendix*, Fig. S3 *B* and *C* and Table S2). Repressed proteins were enriched with digestive enzymes (trypsins, proteases, maltases, glucosidases). Next, we decided to conclusively test some of the defense pathways using respective mutants.

Considering the important role of the IMD pathway in the defense against *P. entomophila* gut infection (40) and that the IMD pathway was upregulated in both sexes (Fig. 2 *A* and *C* and *SI Appendix*, Fig. S3*A*), we tested the contribution of this pathway to sexual dimorphism. Although *Relish* mutants, deficient in the IMD pathway, were more susceptible to *P. entomophila* infection, they still exhibited sexual dimorphism. Thus, the IMD pathway is important in defense against *P. entomophila,* however, it is not sufficient to explain the observed sexual dimorphism (Fig. 2 *D* and *D’*). Since the peritrophic matrix was described as an important defense barrier against *P. entomophila* infection in female flies (19, 20), we tested whether sex differences in this physical barrier could explain the differences in survival. We infected *Drosocrystallin* (here referred to as *Cry*) loss-of-function mutant deficient in the peritrophic matrix and still observed sexual dimorphism (Fig. 2 *E* and *E’*). Last, we tested whether sexual dimorphism in the Toll pathway is connected to the sex difference in survival after *P. entomophila* infection. We tested three loss-of-function mutants in Toll pathway: *spätzle* (*spz*), *modular serine protease* (*modSP*), and *MyD88*. All mutants exhibited sex differences in survival comparable to wild-type flies (*SI Appendix*, Fig. S3 *D*–*F’*), indicating that the Toll pathway does not underlie the observed sexual dimorphism.

### *P. entomophila* Causes Sex-Biased Defecation Blockage.

**Fig. 3.**
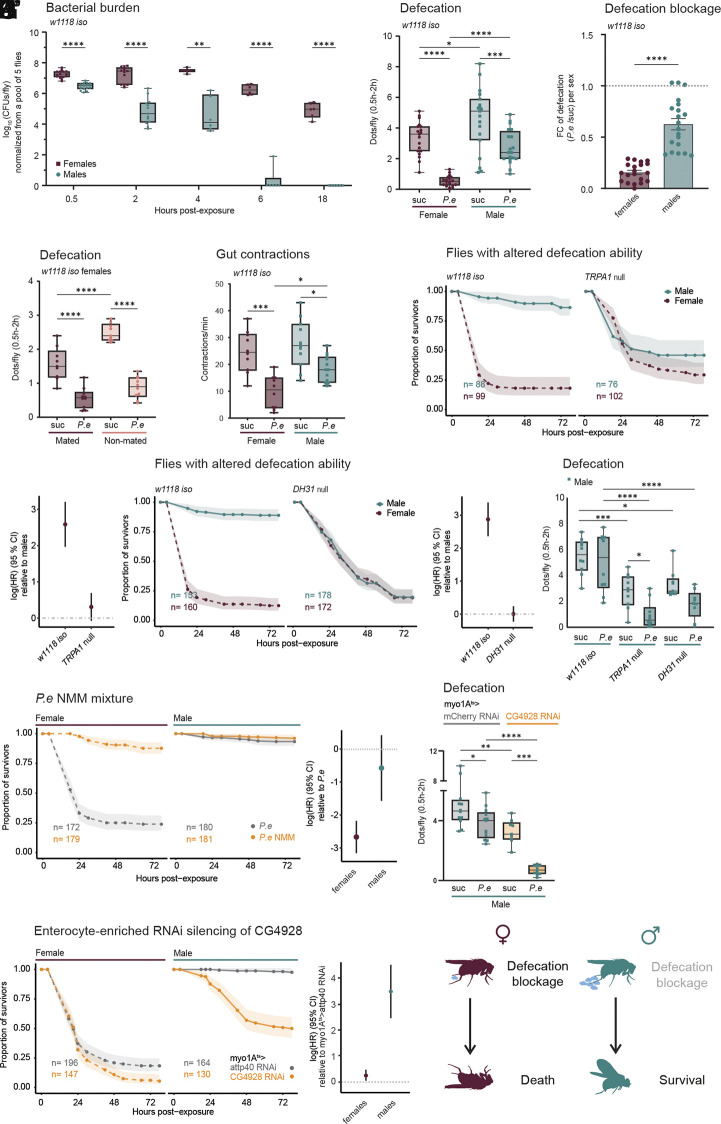
*P. entomophila* causes sex-biased defecation blockage. (*A*) Pathogen abundance (CFUs) at different timepoints postexposure to *P. entomophila* in 0.5 h protocol. One dot indicates biological replicate (pool of five flies). Significance by Mixed-effects model (REML) with Šídák’s multiple comparisons test for each time point. (*B* and *B’*) The defecation rate of female and male *w^1118^* iso flies measured 0.5 to 2 h after exposure to blue-dyed sucrose (control) or *P. entomophila* in 0.5 h protocol. (*B*) Significance by two-way ANOVA with Šídák’s multiple comparisons test. Interaction (Sex × Treatment) *P* = n.s (0.0947). (*B’*) Fold change calculated from mean values of sucrose control of appropriate sex from the same biological repeat. Significance by the Mann–Whitney test. (*C*) The defecation rate of mated and virgin female *w^1118^* iso flies measured 0.5 to 2 h after exposure to blue-dyed sucrose (control) or *P. entomophila* in 0.5 h protocol. Significance by ordinary one-way ANOVA, Šídák’s multiple comparisons test. (*D*) Gut contraction count of *w^1118^* iso flies after exposure to sucrose (control) or *P. entomophila* in 1 h protocol using ex vivo assay. Significance by ordinary one-way ANOVA, Šídák’s multiple comparisons test. (*E*–*F’*) Survival curves with 95% CI (shaded area) and hazard ratios with 95% CI of *w^1118^* iso (control) and two mutants with impaired defecation ability: (*E* and *E’*) *TRPA1* loss-of-function mutant and (*F* and *F’*) *DH31* loss-of-function mutant. (*G*) The defecation rate of male *w^1118^* iso (control) flies and two mutants with impaired defecation ability (*TRPA1* loss-of-function mutant and *DH31* loss-of-function mutant) measured 0.5 to 2 h after exposure to blue-dyed sucrose (control) or *P. entomophila* in 0.5 h protocol. Significance by ordinary one-way ANOVA, Šídák’s multiple comparisons test. (*H* and *H’*) Survival curves with 95% CI (shaded area) and hazard ratios with 95% CI of *w^1118^* iso flies upon exposure to *P. entomophila* and *P. entomophila*/N-methyl maleimide mixture. (*I* and *I’*) Survival curves with 95% CI (shaded area) and hazard ratios with 95% CI of *myo1A^ts^>attp40* RNAi (control) and *myo1A^ts^>CG4928* RNAi upon exposure to *P. entomophila*. (*J*) The defecation rate of male *myo1A^ts^>attp40* RNAi (control) and *myo1A^ts^>CG4928* RNAi flies measured 0.5 to 2 h after exposure to blue-dyed sucrose (control) or *P. entomophila* in 0.5 h protocol. Significance by two-way ANOVA with Šídák’s multiple comparisons test. Interaction (Genotype × Treatment) *P* = n.s (0.1570). (*K*) Graphical summary illustrating the link between sexual dimorphism in defecation and survival. Throughout the panels, boxplots and dot plots show median and interquartile ranges (IQR); whiskers show the full data range. *P < 0.05, **P < 0.01, ***P < 0.001, and ****P < 0.0001.

*Drosophila* survivorship following infection is determined by the interplay of immune activity and pathogen burden at critical time points (49). Therefore, we measured *P. entomophila* load in infected flies to assess whether males and females differ in the ability to control pathogens. To reflect only the behavior of bacteria in the gut and not the influence of possible reingestion of bacteria, bacterial load was measured using flies exposed to *P. entomophila* for 0.5 h [which has a similar effect on survival as 24 h infections (Fig. 1*J* and *SI Appendix*, Fig. S2*E*)]. While the *P. entomophila* burden remained high through different time points, as previously described in female flies (39), male flies started clearing *P. entomophila* already at early time points (2 h), and completed pathogen clearance approximately 6 h postexposure (Fig. 3*A*).

Next, we investigated how males clear the pathogen: via killing in the intestine or expulsion through gut peristalsis. To test the pathogen clearance via expulsion by gut peristalsis, we infected flies with a mixture of *P. entomophila* and blue color dye that allowed to estimate the intestinal excretion by scoring the number of defecation spots deposited within 1.5 h after a 0.5 h exposure (31). We found that *P. entomophila* significantly reduced defecation in both males and females as compared to sucrose controls (Fig. 3*B*). However, the effect was much stronger in females, leading to almost complete defecation blockage (Fig. 3*B’*). Defecation blockage after infection was also observed in virgin flies (Fig. 3*C*), confirming that the sex differences are not confounded by mating status. We directly examined gut contractions in infected male and female flies using an ex vivo assay. We observed higher contraction rates in male guts as compared to female guts after *P. entomophila* infection (Fig. 3*D* and Movies S1–S4), suggesting that reduced gut peristalsis is likely responsible for reduced defecation frequency in females. We genetically and chemically manipulated defecation frequency during *P. entomophila* infection to test the causality between defecation and survivorship. Upon bacterial ingestion, the gut produces ROS sensed by evolutionarily conserved Transient Receptor Potential A1 channel (TRPA1) in enteroendocrine cells that, as a result, release Diuretic Hormone 31 (DH31), which activates gut contraction favoring pathogen expulsion (31, 32). When we compared mutants lacking *TRPA1* or *DH31*, the sexual dimorphism in susceptibility was not present anymore; males were as susceptible as females to *P. entomophila* (Fig. 3 *E***–***F’*) and they had reduced defecation in comparison to wild-type control (Fig. 3*G*). We then tested whether increasing defecation decreased susceptibility by exposing infected flies of both sexes to N-methyl maleimide (NMM), a chemical that increases gut contractions by activating TRPA1 (31). The low susceptibility of males was not affected. However, it reduced strongly the female susceptibility, bringing them close to those of males (Fig. 3*H* and *H’*).

We investigated the genetic basis of the susceptibility to *P. entomophila* oral infection in each sex (*SI Appendix*, Fig. S1 *A* and *B*), and of their dimorphism (*SI Appendix*, Fig. S1*C*) using a GWAS. To do so, we used the genetic and phenotypic variations present in each sex in the DGRP lines and in *w^1118^* iso, the latter being used as a reference across the experiments. The candidate SNPs associated with male susceptibility, female susceptibility, and sexual dimorphism in susceptibility are listed in *SI Appendix*, Table S3 and curated in *SI Appendix*, Fig. S4 *A*–*C* based on the functional impact of the mutation (i.e., missense, UTR, within gene or intron/splice region), the *P* value, and the size of the effects observed on the DGRP lines [i.e., log (hazard ratio)].

Then, to validate the potential contribution of those SNPs to variation in susceptibility to *P. entomophila*, we tested whether the change in expression of genes associated with those SNPs will result in a difference in susceptibility. We were interested to find those candidates affecting the processes during the infection; therefore, we targeted the expression of some candidate genes (listed in *SI Appendix*, Table S3) specifically in enterocytes at the adult stage (*SI Appendix*, Fig. S5 *A* and *A’*). The strongest effect on sexual dimorphism in survival was observed when we reduced enterocyte expression of *CG4928* (Fig. 3 *I* and *I’* and *SI Appendix*, Fig. S5 *B* and *B’*). *CG4928* is an understudied transporter predicted to enable potassium channel regulator activity and transmembrane transport of potassium (50). Considering that *CG4928* shows high expression level in the gut (51) and known link between potassium and intestinal motility (52, 53), we hypothesized that *CG4928* affects susceptibility to infection via altering pathogen clearance by defecation. Indeed, we found that while control males still retained defecation ability after infection, in *CG4928* RNAi male’s defecation was almost completely inhibited (Fig. 3*J*). Hence, our GWAS approach provided an additional unbiased confirmation of the link between survival and defecation rates.

Altogether, these results support a scenario where males, due to their reduced susceptibility to *P. entomophila*-induced defecation blockage, can efficiently clear the pathogen via gut peristalsis and thus survive the infection (Fig. 3*K*).

### Male Gut Antioxidant Capacity Contributes to the Attenuated Impact of *P. entomophila* on Defecation Blockage.

**Fig. 4.**
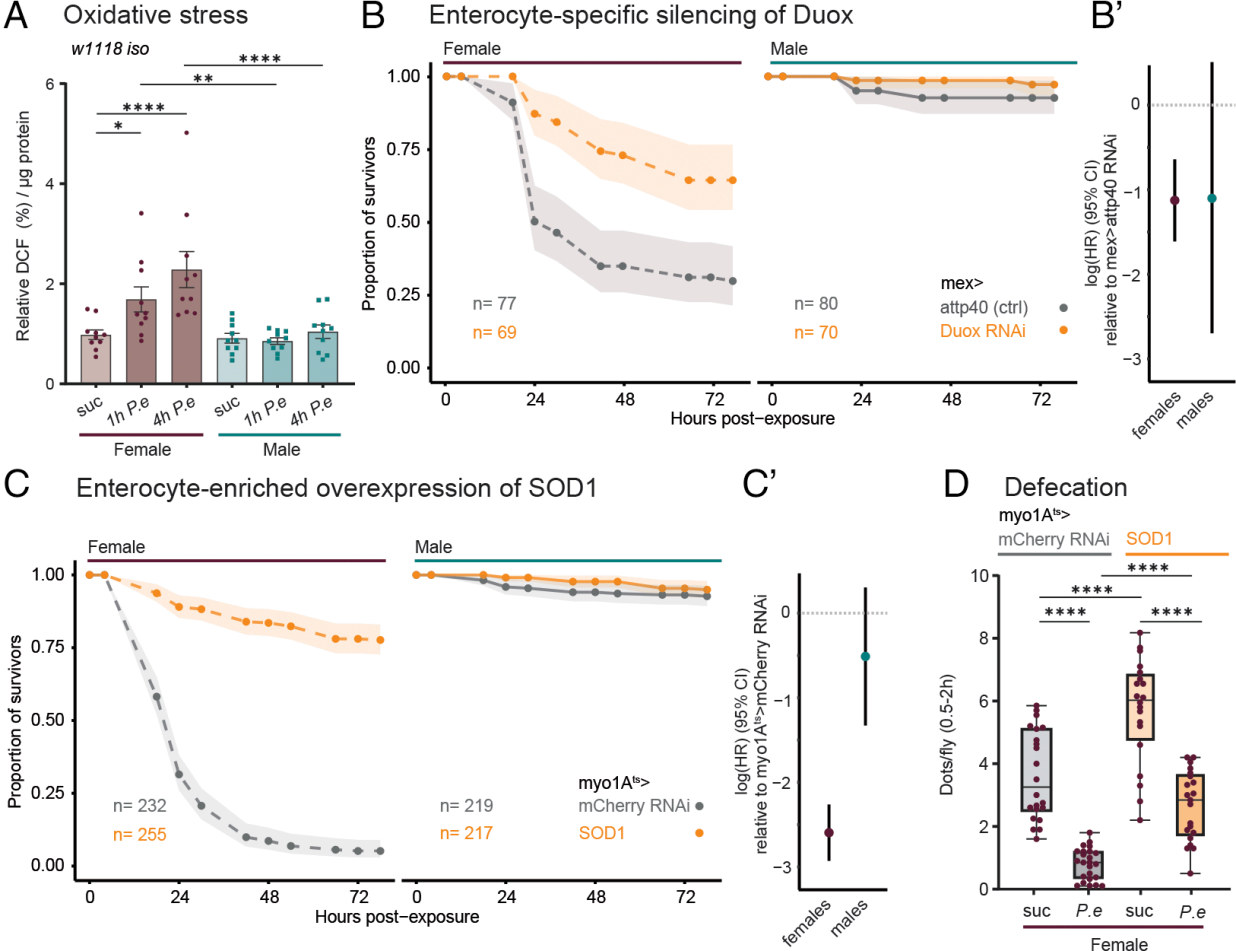
Male gut antioxidant capacity contributes to the attenuated impact of *P. entomophila* on defecation blockage. (*A*) Reactive oxygen species measured as percent of 2′,7′-dichlorofluorescein (DCF) relative fluorescence units (RFU) normalized per protein of homogenized gut samples. (N = 10 samples, n = 30 guts per sample). Mean ± SE. Significance by two-way ANOVA with Šídák’s multiple comparisons test. Interaction (Sex × Treatment) *P* = * (0.0152). (*B* and *B’*) Survival curves with 95% CI (shaded area) and hazard ratios with 95% CI of female and male *mex>attp40 RNAi* (control) and *mex>duox RNAi* (BL38907). (*C* and *C’*) Survival curves with 95% CI (shaded area) and hazard ratios with 95% CI of female and male *myo1A^ts^>mCherry* RNAi (control) and *myo1A^ts^>UAS-SOD1* (BL24750). (*D*) The defecation of female *myo1A^ts^>mCherry* RNAi (control) and *myo1A^ts^>UAS-SOD1* (BL24750) flies measured 0.5 to 2 h after exposure to blue-dyed sucrose (control) or *P. entomophila* in 0.5 h protocol. Significance by two-way ANOVA with Šídák’s multiple comparisons test. Interaction (Genotype × Treatment) *P* = n.s (0.6911). *P < 0.05, **P < 0.01, ***P < 0.001, and ****P < 0.0001.

Considering the known role of ROS in gut peristalsis (30–32), we investigated whether male and female differences in ROS production or sensitivity contribute to the observed differences in survival and pathogen eviction via defecation. Given that in female flies, *P. entomophila* induces a high level of intestinal ROS (26, 41), we hypothesized that males might prevent or resist such excessive oxidative stress and thus survive the infection. To compare oxidative stress levels during infection in male and female flies, we measured 2’,7’-dichlorofluorescein (H_2_DCF), which reflects general oxidative stress levels (54). Consistent with previous studies (26, 41), we detected a significant increase in oxidative stress in female guts after *P. entomophila* ingestion (Fig. 4*A*). Such an increase was not observed in male flies (Fig. 4*A*). We confirmed this finding by measuring the level of hydrogen peroxide using a fluorometric hydrogen peroxidase assay (*SI Appendix*, Fig. S6*A*). Because of the correlation between oxidative stress and susceptibility to *P. entomophila* infection (41), we wondered whether male flies have higher antioxidant capacity by exposing flies to commonly used oxidizing agent paraquat (26). Male flies were less susceptible to paraquat than females (*SI Appendix*, Fig. S6 *B* and *B’*), indicating they can handle oxidative stress better.

Then, we wondered whether sex differences in *P. entomophila-*induced oxidative stress contribute to observed differences in survival. We tested the effect of N-acetyl-L-cysteine (NAC), a commonly used antioxidant compound (55). We observed that both infected female and male flies survived significantly longer when, after infection, they were fed NAC-supplemented sucrose as compared to sucrose alone (*SI Appendix*, Fig. S6 *C* and *C’*). To identify the source of excessive ROS, we knocked down by RNAi *Nox* and *Duox*—two enzymes previously implicated in ROS generation in response to bacteria (26, 28, 56). While *Nox* knockdown with two different RNAi lines had no effect on fly susceptibility to *P. entomophila* (*SI Appendix*, Fig. S6 *D*–*E’*), females of two tested *Duox* RNAi lines exhibited increased survival to infection (Fig. 4 *B* and *B’* and *SI Appendix*, Fig. S6 *F* and *F’*). Our RNA-seq detected low and variable expression of *Duox* transcripts in the gut with a trend toward higher expression in females, particularly at 16 h post–*P. entomophila* infection (*SI Appendix*, Fig. S6*G*). To test whether Duox is involved in the excessive ROS generation in females, we quantified ROS levels in the enterocyte-specific *Duox* RNAi line. H2DCF-DA–based assay did not show differences between control and *Duox* RNAi lines at 1 h postinfection and detected even higher ROS in *Duox* RNAi at 4 h post–*P. entomophila* infection (*SI Appendix*, Fig. S6*H*). Hydrogen peroxide kit similarly did not detect reduced ROS levels in *Duox* RNAi line (*SI Appendix*, Fig. S6*I*). Thus, Duox is not required for an excessive ROS generation in females.

Additionally, we tested whether the expression of antioxidant enzymes in enterocytes affects fly susceptibility to infection. We observed an increase in survival of infected flies with overexpression of cytosolic antioxidant enzyme superoxide dismutase 1 (SOD1) with two distinct transgenic lines and GAL4 drivers (Fig. 4 *C* and *C’* and *SI Appendix*, Fig. S6 *J*–*K’*) and a minimal effect of overexpression of mitochondrial SOD2 (*SI Appendix*, Fig. S6 *L* and *L’*). Additionally, we fed SOD1-overexpressing female flies with *P. entomophila,* paraquat, or a mixture of *P. entomophila* and paraquat to test whether we can override the antioxidant capacity of SOD1 with paraquat. Although SOD1-overexpressing flies survived better on *P. entomophila* or paraquat alone compared to control flies, they could not survive the excessive oxidative stress caused by the *P. entomophila/*paraquat mixture (*SI Appendix*, Fig. S6 *M* and *M’*). Overall, these results indicate that cytosolic (and not mitochondrial) oxidative stress is a determinant of susceptibility to infection in female flies, and differences between sexes in oxidative stress contribute to sexual dimorphism in susceptibility to *P. entomophila* gut infection.

To further reinforce the relationship between oxidative stress, susceptibility to infection, and newly described defecation blockage, we tested whether altering oxidative stress genetically or chemically will also affect the defecation. Overexpression of SOD1 in female flies also significantly increased defecation after infection (Fig. 4*D*), suggesting that reducing excessive ROS in females restores gut peristalsis and pathogen clearance. When we increased oxidative stress in males by combining *P. entomophila* with paraquat, we observed not only high susceptibility of males to *P. entomophila* (mixed with paraquat) infection (*SI Appendix*, Fig. S7 *A* and *A’*) but also significantly reduced defecation (*SI Appendix*, Fig. S7*B*), suggesting that the reduced ability to clear the pathogen affects survival. Males’ ability to prevent the infection-induced oxidative burst allows them to clear the pathogen via intestinal peristalsis and survive the infection (*SI Appendix*, Fig. S7*C*).

### Abolishing Differences in NADPH Metabolism Removes Sexual Dimorphism in Susceptibility to Infection.

**Fig. 5.**
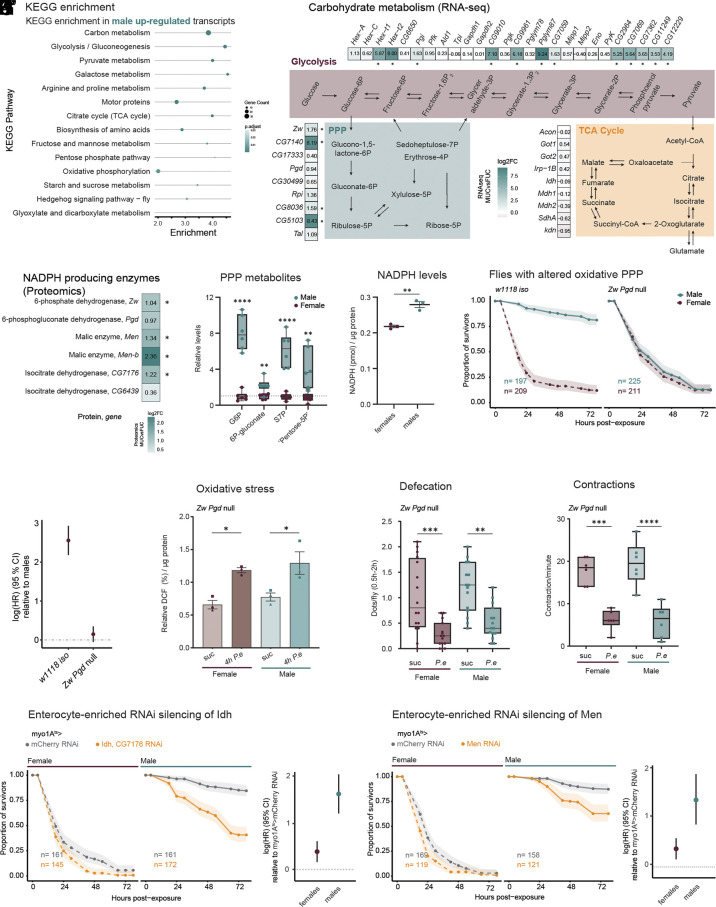
Abolishing differences in NADPH metabolism removes sexual dimorphism in susceptibility to infection. (*A*) KEGG pathway analysis of genes differentially regulated between male and female guts under sucrose-fed (control) conditions. (*B*) The schematic of glycolysis, pentose phosphate pathway (PPP) and tricarboxylic acid (TCA) cycle with respective heatmap showing expression differences (log_2_FC) of selected genes between male and female sucrose-fed guts. Significance (padj < 0.1, |log_2_FC| > 1.5) indicated by *. (*C*) Heatmap showing abundance difference (log_2_FC) of NADPH producing enzymes between male and female sucrose-fed guts. Significance (padj < 0.05, |log2FC| > 1) indicated by *. (*D*) Dot plots show metabolite levels normalized to the total protein content of whole-body homogenates relative to mean of female control. Significance by unpaired Student’s *t* test. (*E*) Dot plots show the NADPH level of five pooled whole-body homogenates normalized to protein concentration. Mean ± SEM (N = 3). Significance by unpaired Student’s *t* test. (*F* and *F’*) Survival curves with 95% CI (shaded area) and hazard ratios with 95% CI of *w^1118^* iso (control) flies and *Zw Pgd* double null mutant flies upon exposure to *P. entomophila*. (*G*) Reactive oxygen species measured as percent of 2′,7′-dichlorofluorescein (DCF) relative fluorescence units (RFU) normalized per protein of homogenized gut samples. Data were normalized per female sucrose sample. (N = 3 independent samples, n = 30 guts per sample). Time points: sucrose and 4 h post–*P. entomophila* infection. Mean ± SE. Significance by two-way ANOVA with Tukey’s multiple comparisons test. Interaction (Sex × Treatment) *P* = n.s. (0.9752). (*H*) The defecation of female and male *Zw Pgd* double null mutant flies measured 0.5 to 2 h after exposure to blue-dyed sucrose (control) or *P. entomophila* in 0.5 h protocol. Significance by two-way ANOVA with Šídák’s multiple comparisons test. Interaction (Sex × Treatment) *P* = n.s (0.8060). (*I*) Gut contraction count of *w^1118^* iso flies after exposure to sucrose (control) or *P. entomophila* in 1 h protocol using ex vivo assay. Significance by ordinary one-way ANOVA, Šídák’s multiple comparisons test. (*J*–*K’*) Survival curves with 95% CI (shaded area) and hazard ratios with 95% CI of female and male *myo1A^ts^>mCherry* RNAi (control) and: (*J* and *J’*) *myo1A^ts^>Idh*, *CG7176* RNAi (flies with knockout of NADPH producing enzyme Idh in the enterocytes), (*K* and *K’*) *myo1A^ts^>Men* RNAi (flies with knockout of NADPH producing enzyme Men in the enterocytes) upon exposure to *P. entomophila*. *P < 0.05, **P < 0.01, ***P < 0.001, and ****P < 0.0001.

Next, we wanted to understand the mechanisms that mediate males’ resistance to oxidative stress. The transcriptomic and proteomic analyses in both uninfected sexes (*SI Appendix*, Table S4) showed male-biased upregulation of genes involved in carbohydrate metabolism. This was suggested by the functional enrichment analysis of transcripts in male intestines (Fig. 5*A*) and is consistent with a previous study (43). In particular, numerous transcripts encoding steps in glycolysis, the pentose phosphate pathway (PPP), and the tricarboxylic acid cycle were expressed stronger in males (Fig. 5 *B* and *C*). The proteomic analysis confirmed that male intestines have increased abundance of proteins involved in carbohydrate metabolism than females (*SI Appendix*, Fig. S8 *A* and *B*). To illustrate that these higher levels result in a higher pathway activity in males, we performed targeted measurement of key intermediate metabolites of PPP. We detected a significantly higher relative amount of metabolites in male flies (Fig. 5*D*), which suggests a higher metabolic flux through the PPP in males. Consistent with this, NADPH—a key product of PPP was more abundant in males than in females (Fig. 5*E*). Given the prominent role of PPP in the antioxidant defense (57, 58), we hypothesized that elevated PPP activity in males contributes to their resistance to oxidative stress and, thus, to *P. entomophila* infection. To test this hypothesis, we used double mutant *Pgd Zw*, which lacks functional glucose 6-phosphate dehydrogenase (*Zwischenferment* or *Zw*), the first enzyme in the oxidative PPP, and 6-phosphogluconate dehydrogenase (*Pgd*), the third enzyme in the oxidative PPP (59). Survival analysis showed that *Pgd Zw* mutant males were as sensitive as females to *P. entomophila* infection (Fig. 5 *F* and *F’*), had high level of ROS (Fig. 5*G*) and exhibited intestinal transit blockage (Fig. 5*H*), which correlated with reduced gut contractions (Fig. 5*I*). Thus, PPP is necessary for males to resist *P. entomophila* infection and to clear the pathogen via intestinal peristalsis.

Considering that NADPH is a key metabolite of PPP mediating protection against oxidative stress via the production of antioxidants, such as glutathione, we tested the role of additional NADPH-producing enzymes during *P. entomophila* infection. Besides Zw and Pgd, the two enzymes of PPP, the NADPH metabolic network in *Drosophila* includes cytosolic malate dehydrogenase (Malic enzyme, Men), mitochondrial malate dehydrogenase (Malic enzyme b, Men-b), cytosolic isocitrate dehydrogenase (Idh, CG7176), and mitochondrial isocitrate dehydrogenase (Idh, CG6439) (60). Among these, Zw and Men-b were expressed stronger in male guts when comparing transcripts (*SI Appendix*, Fig. S8*C*), but all of them (except Pgd and mitochondrial Idh, CG6439) had higher protein levels (Fig. 5*C*). Enterocyte-specific knockdown of cytosolic Idh (CG7176) and Men by RNAi resulted in increased susceptibility of males to *P. entomophila* infection (Fig. 5 *J*–*K’* and *SI Appendix*, Fig. S8 *E*–*F’*). Knockdown of mitochondrial Idh CG6439 had no significant effect on survival (*SI Appendix*, Fig. S8 *D* and *D’*). Thus, cytosolic NADPH-producing enzymes are required for increased survival of males to *P. entomophila* infection, likely by providing protection against oxidative stress and consequent suppression of pathogen clearance via gut peristalsis.

### *P. entomophila* Virulence Regulator Hfq Is Expressed Higher in Female Guts.

**Fig. 6.**
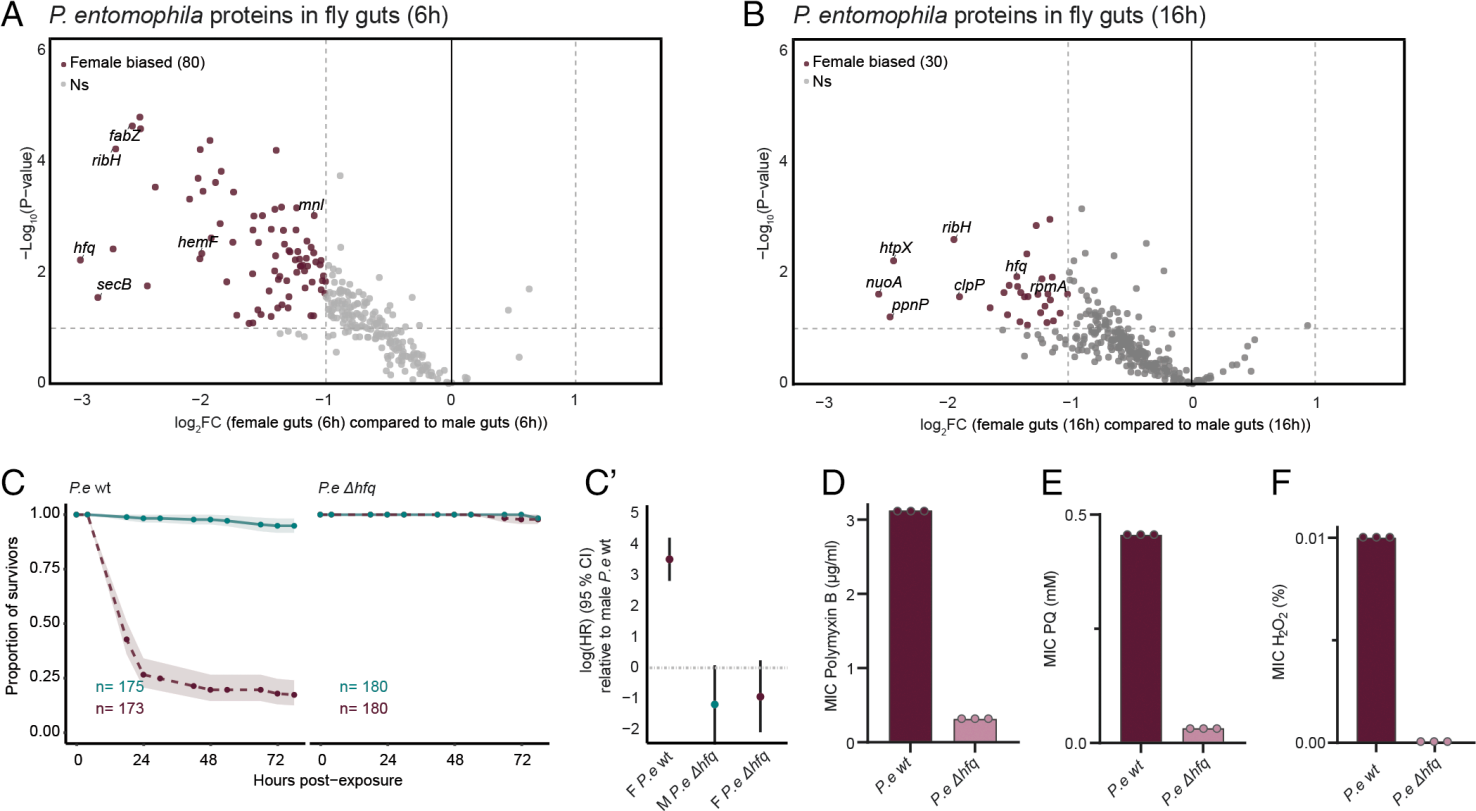
*P. entomophila* virulence regulator Hfq is expressed higher in female guts. (*A* and *B*) Volcano plots of differentially abundant *P. entomophila* proteins (│log_2_FC│≥ 1 and padj cut-off 0.05) between infected male and female guts (*A*) 6 h and (*B*) 16 h postexposure to *P. entomophila* (N = 5 samples, each with 30 guts). (*C* and *C’*) Survival curves with 95% CI (shaded area) and hazard ratios with 95% CI upon exposure to wild-type *P. entomophila* and *P. entomophila Δhfq*. (*D*–*F*) Polymyxin B (*D*), paraquat (*E*), or hydrogen peroxide (*F*) minimum inhibitory concentration values for wild-type and *P. entomophila Δhfq* mutant (N = 3).

To test whether a sexually dimorphic gut environment results in a sex-specific pathogen response, we undertook a proteomics approach to identify proteins differentially produced by *P. entomophila* in the guts of each sex. We detected several *P. entomophila* proteins that differed significantly in abundance between male and female guts (Fig. 6 *A* and *B* and *SI Appendix*, Table S5). Among these proteins, RNA chaperon Hfq, which has a role in pathogen virulence in other studies (61, 62), was the protein with the largest difference at 6 h (*SI Appendix*, Table S5). We hypothesized that higher expression of Hfq in female flies would result in higher *P. entomophila* virulence, and consequently lower survival of female flies.

To test whether Hfq plays a role in *P. entomophila* virulence in *Drosophila*, we infected flies with the *P. entomophila* ∆*hfq* mutant (63). Survival analysis showed that the ∆*hfq* mutant was avirulent, and both male and female flies survived the infection (Fig. 6 *C* and *C’*). Given the prominent role of Hfq in bacterial stress response (64), we wondered whether Hfq contributed to the bacterial evasion of female *Drosophila* immune effectors such as ROS and AMPs. To test this, we performed minimum inhibitory concentration (MIC) assay with compounds that should mimic the action of ROS and AMPs (65–68). The *P. entomophila ∆hfq* mutant was more sensitive to all compounds that we tested: polymyxin B (Fig. 6*D*), paraquat (Fig. 6*E*), or hydrogen peroxide (H_2_O_2_) (Fig. 6*F*), suggesting that Hfq is necessary for bacterial ability to counteract host immune effectors. Overall, these findings suggest that the crucial regulator of virulence and stress response Hfq, is expressed higher in the female gut environment, contributing to female-biased virulence of *P. entomophila*.

## Discussion

We report differences between male and female *Drosophila* in susceptibility to intestinal infection. Our results support the following model (*SI Appendix*, Fig. S9). Upon ingestion, *P. entomophila* induces a strong oxidative response in female guts, leading to defecation blockage, pathogen persistence, and death of the flies due to gut damage. However, our data indicate that overall ROS levels alone do not explain female susceptibility. Rather, susceptibility is linked to the source and regulation of ROS production, including Duox-dependent processes. Male flies overcome the pathogen-induced oxidative burst due to elevated basal activity of a key antioxidant system—PPP. This allows males to retain intestinal transit during infection which expels the pathogens. We further showed that the pathogen produced more of the virulence regulator Hfq in female guts. Our work not only uncovers the mechanistic underpinnings of sexual dimorphism in *Drosophila* susceptibility to intestinal infection but also expands our understanding of virulence strategies utilized by intestinal pathogens.

Our study identified sex differences in the defecation frequency as a key factor explaining the sexual dimorphism in pathogen load and fly survival. Pathogen clearance via defecation is a conserved defense mechanism against intestinal infections (69–71). In *Drosophila*, ROS production activates TRPA1-DH31 signaling cascade to initiate gut peristalsis (31, 32). Given that ROS is necessary to promote defecation (31), one would anticipate that higher ROS in females would trigger higher defecation, however this is not what we observed. Our data indicate that bulk ROS measurements may obscure functionally distinct contributions of different ROS sources. Indeed, enterocyte-specific *Duox* silencing did not abolish the infection-induced ROS increase, yet significantly reduced female susceptibility, suggesting that Duox-dependent processes contribute to pathology independently of total ROS levels. This supports a model in which the source, localization, or chemical nature of ROS is critical. Determining the spatial origin and identity of ROS will be essential in future work, although infection-induced tissue damage currently limits such analyses. How *P. entomophila* triggers dysregulated ROS production in the gut remains an open question. Our RNA-seq data are consistent with a possible induction of *Duox* expression in females at later stages of infection that is not observed in males, raising the possibility that sex-specific regulation of Duox contributes to differential outcomes. Whether this regulation is itself ROS-dependent or linked to other signaling pathways remains to be determined. While there is controversy regarding Duox function, specifically whether Duox-produced ROS are really bactericidal (72), our results are more consistent with a signaling role of Duox than with gut damage caused by Duox-mediated ROS activity.

Using a dual-proteomics approach, we were able to obtain unique insights into the pathogen response to the host environment. Specifically, we detected higher abundance of several *P. entomophila* proteins in female flies. Among these proteins, we further characterized the RNA chaperon Hfq—a known virulence regulator (73). We showed that *P. entomophila ∆hfq* mutant was avirulent to flies and exhibited increased susceptibility to oxidative stress and antimicrobials mimicking the action of *Drosophila* AMPs. Thus, *P. entomophila ∆hfq* mutant exhibits typical phenotypes of attenuated virulence and susceptibility to host defense mechanisms previously reported for *hfq* mutants in other bacteria (61, 64, 74). Since we detected decreased abundance of Hfq protein in male guts, this likely leads to decreased pathogen virulence and ability to resist host immune effectors further contributing to the survival of male flies. Our study uncovered an example when gut metabolic environment not only affects the host defenses against the pathogen but also modulates the pathogen virulence.

## Materials and Methods

Data used to prepare graphs are summarized in *SI Appendix*, Table S6. *Drosophila* strains used in this study are listed in *SI Appendix*, Table S7.

### Oral Infection and Survival Assay.

Unless otherwise indicated, the previously established protocol (Fig. 1*A*) was used for the oral infection of flies (26).

### Metabolites Comparison.

Five whole flies per sample were used following the 6 h sucrose-feeding (control that was used for RNAseq and proteomics). To extract the metabolites, flies were homogenized using a Precellys homogenizer (6,000 rpm, 30 s) in a mixture of 100 µL of 50% methanol and 150 µL of chloroform. Following centrifugation (10,000 g, 20 min, 4 °C), 50 µL of the upper aqueous layer were transferred into HPLC/GC certified vials (Fisherbrand™) on ice. Protein concentration was measured as described above (Pierce™ 660 nm Protein Assay Reagent). Rest of the samples were kept at −80 °C before being analyzed by the Core facility for metabolomics and small molecules mass spectrometry. The parameter settings of all targets are given in *SI Appendix*, Table S8.

Detailed sample sizes and statistical analyses are given in *SI Appendix*, Table S9.

All materials and methods may be found in *SI Appendix*.

## Supplementary Material

Appendix 01 (PDF)

Dataset S01 (XLSX)

Dataset S02 (XLSX)

Dataset S03 (XLSX)

Dataset S04 (XLSX)

Dataset S05 (XLSX)

Dataset S06 (XLSX)

Dataset S07 (XLSX)

Dataset S08 (XLSX)

Dataset S09 (XLSX)

Movie S1. Representative movie of a female *w^1118^* iso gut showing contractions 1 h after sucrose feeding, recorded in Schneider’s medium. The video represents a 1 min period. Video duration: 30 s (2 frames per second).

Movie S2. Representative movie of a female *w^1118^* iso gut showing contractions 1 h after exposure to *P. entomophila*, recorded in Schneider’s medium. The video represents a 1 min period. Video duration: 30 s (2 frames per second).

Movie S3. Representative movie of a male *w^1118^* iso gut showing contractions 1 h after sucrose feeding, recorded in Schneider’s medium. The video represents a 1 min period. Video duration: 30 s (2 frames per second).

Movie S4. Representative movie of a male *w^1118^* iso gut showing contractions 1 h after exposure to *P. entomophila*, recorded in Schneider’s medium. The video represents a 1 min period. Video duration: 30 s (2 frames per second).

## Data Availability

The mass spectrometry proteomics data have been deposited to the ProteomeXchange Consortium via the PRIDE partner repository with the dataset identifier PXD064190 (75). RNA-seq dataset is available through the National Center for Biotechnology Information (NCBI) Sequence Read Archive (SRA) under accession number PRJNA1256345 (76). Other data are included in the article and/or supporting information.

## References

[1] C. Ober, D. A. Loisel, Y. Gilad, Sex-specific genetic architecture of human disease. Nat. Rev. Genet. **9**, 911–922 (2008). 19002143 10.1038/nrg2415 PMC2694620

[2] J. W. Millington, E. J. Rideout, Sex differences in Drosophila development and physiology. Curr. Opin. Physiol. **6**, 46–56 (2018).

[3] R. L. Belmonte, M.-K. Corbally, D. F. Duneau, J. C. Regan, Sexual dimorphisms in innate immunity and responses to infection in Drosophila melanogaster. Front. Immunol. **10**, 3075 (2020). 32076419 10.3389/fimmu.2019.03075 PMC7006818

[4] S. L. Klein, K. L. Flanagan, Sex differences in immune responses. Nat. Rev. Immunol. **16**, 626–638 (2016). 27546235 10.1038/nri.2016.90

[5] M. M. Khan, R. M. Graze, Sex dimorphism in expression of immune response genes in Drosophila. bioRxiv [Preprint] (2024). 10.1101/2024.07.27.605461 (Accessed 19 September 2024).

[6] L. G. vom Steeg, S. L. Klein, SeXX matters in infectious disease pathogenesis. PLoS Pathog. **12**, e1005374 (2016). 26891052 10.1371/journal.ppat.1005374 PMC4759457

[7] D. F. Duneau , The Toll pathway underlies host sexual dimorphism in resistance to both Gram-negative and Gram-positive bacteria in mated Drosophila. BMC Biol. **15**, 124 (2017). 29268741 10.1186/s12915-017-0466-3 PMC5740927

[8] D. Duneau, D. Ebert, Host sexual dimorphism and parasite adaptation. PLoS Biol. **10**, e1001271 (2012). 22389630 10.1371/journal.pbio.1001271 PMC3289593

[9] D. Duneau, P. Luijckx, L. F. Ruder, D. Ebert, Sex-specific effects of a parasite evolving in a female-biased host population. BMC Biol. **10**, 104 (2012). 23249484 10.1186/1741-7007-10-104 PMC3568004

[10] F. Úbeda, V. A. A. Jansen, The evolution of sex-specific virulence in infectious diseases. Nat. Commun. **7**, 13849 (2016). 27959327 10.1038/ncomms13849 PMC5159935

[11] S. P. Dias, M. C. Brouwer, D. van de Beek, Sex and gender differences in bacterial infections. Infect. Immun. **90**, e00283-22 (2022). 36121220 10.1128/iai.00283-22 PMC9584217

[12] M. Elderman , Sex and strain dependent differences in mucosal immunology and microbiota composition in mice. Biol. Sex Differ. **9**, 26 (2018). 29914546 10.1186/s13293-018-0186-6 PMC6006852

[13] N. Buchon, N. Silverman, S. Cherry, Immunity in Drosophila melanogaster—from microbial recognition to whole-organism physiology. Nat. Rev. Immunol. **14**, 796–810 (2014). 25421701 10.1038/nri3763 PMC6190593

[14] H. Westlake, M. A. Hanson, B. Lemaitre, The Drosophila Immunity Handbook (EPFL Press, 2024).

[15] J. C. Regan , Sex difference in pathology of the ageing gut mediates the greater response of female lifespan to dietary restriction. Elife **5**, e10956 (2016). 10.7554/eLife.10956 PMC4805549 26878754

[16] T. F. Paulo, P. A. Akyaw, T. Paixão, É. Sucena, Evolution of resistance and disease tolerance mechanisms to oral bacterial infection in Drosophila melanogaster. Open Biol. **15**, 240265 (2025). 40068814 10.1098/rsob.240265 PMC11896704

[17] J. A. Siva-Jothy, P. F. Vale, Dissecting genetic and sex-specific sources of host heterogeneity in pathogen shedding and spread. PLoS Pathog. **17**, e1009196 (2021). 33465160 10.1371/journal.ppat.1009196 PMC7846003

[18] A. Hrdina, I. Iatsenko, Sex bias in iron sequestration by transferrin 1 modulates sexually dimorphic infection outcomes in Drosophila melanogaster. Genetics **233**, iyag058 (2026), 10.1093/genetics/iyag058. 41766471 PMC13147530

[19] T. Shibata , Crosslinking of a peritrophic matrix protein protects gut epithelia from bacterial exotoxins. PLoS Pathog. **11**, e1005244 (2015). 26506243 10.1371/journal.ppat.1005244 PMC4646701

[20] T. Kuraishi, O. Binggeli, O. Opota, N. Buchon, B. Lemaitre, Genetic evidence for a protective role of the peritrophic matrix against intestinal bacterial infection in Drosophila melanogaster. Proc. Natl. Acad. Sci. U.S.A. **108**, 15966–15971 (2011). 21896728 10.1073/pnas.1105994108 PMC3179054

[21] S. Bai , Bacterial-induced Duox-ROS regulates the Imd immune pathway in the gut by modulating the peritrophic matrix. Cell Rep. **44**, 115404 (2025). 40053451 10.1016/j.celrep.2025.115404

[22] N. Buchon , Morphological and molecular characterization of adult midgut compartmentalization in Drosophila. Cell Rep. **3**, 1725–1738 (2013). 23643535 10.1016/j.celrep.2013.04.001

[23] A. J. Barron , Microbiome-derived acidity protects against microbial invasion in *Drosophila*. Cell Rep. **43**, 114087 (2024). 38583152 10.1016/j.celrep.2024.114087 PMC11163447

[24] G. Storelli , Drosophila Perpetuates nutritional mutualism by promoting the fitness of its intestinal symbiont Lactobacillus plantarum. Cell Metab. **27**, 362–377.e8 (2018). 29290388 10.1016/j.cmet.2017.11.011 PMC5807057

[25] N. Buchon, N. A. Broderick, M. Poidevin, S. Pradervand, B. Lemaitre, Drosophila intestinal response to bacterial infection: Activation of host defense and stem cell proliferation. Cell Host Microbe **5**, 200–211 (2009). 19218090 10.1016/j.chom.2009.01.003

[26] S. Chakrabarti, P. Liehl, N. Buchon, B. Lemaitre, Infection-induced host translational blockage inhibits immune responses and epithelial renewal in the Drosophila gut. Cell Host Microbe **12**, 60–70 (2012). 22817988 10.1016/j.chom.2012.06.001

[27] I. Iatsenko, A. Marra, J. P. Boquete, J. Peña, B. Lemaitre, Iron sequestration by transferrin 1 mediates nutritional immunity in Drosophila melanogaster. Proc. Natl. Acad. Sci. U.S.A. **117**, 7317–7325 (2020). 32188787 10.1073/pnas.1914830117 PMC7132258

[28] K.-A. Lee , Bacterial-derived uracil as a modulator of mucosal immunity and gut-microbe homeostasis in Drosophila. Cell **153**, 797–811 (2013). 23663779 10.1016/j.cell.2013.04.009

[29] E.-M. Ha, C.-T. Oh, Y. S. Bae, W.-J. Lee, A direct role for dual oxidase in Drosophila gut immunity. Science **310**, 847–850 (2005). 16272120 10.1126/science.1117311

[30] F. Tleiss , Spatial and temporal coordination of Duox/TrpA1/Dh31 and IMD pathways is required for the efficient elimination of pathogenic bacteria in the intestine of Drosophila larvae. Elife **13**, RP98716 (2024). 39576741 10.7554/eLife.98716 PMC11584180

[31] E. J. Du , TrpA1 regulates defecation of food-borne pathogens under the control of the duox pathway. PLoS Genet. **12**, e1005773 (2016). 26726767 10.1371/journal.pgen.1005773 PMC4699737

[32] O. Benguettat , The DH31/CGRP enteroendocrine peptide triggers intestinal contractions favoring the elimination of opportunistic bacteria. PLoS Pathog. **14**, e1007279 (2018). 30180210 10.1371/journal.ppat.1007279 PMC6138423

[33] Z. Liu, H. Zhang, B. Lemaitre, X. Li, Duox activation in *Drosophila* Malpighian tubules stimulates intestinal epithelial renewal through a countercurrent flow. Cell Rep. **43**, 114109 (2024). 38613782 10.1016/j.celrep.2024.114109

[34] N. Buchon, N. A. Broderick, S. Chakrabarti, B. Lemaitre, Invasive and indigenous microbiota impact intestinal stem cell activity through multiple pathways in Drosophila. Genes Dev. **23**, 2333–2334 (2009). 19797770 10.1101/gad.1827009 PMC2758745

[35] B. Biteau, C. E. Hochmuth, H. Jasper, Maintaining tissue homeostasis: Dynamic control of somatic stem cell activity. Cell Stem Cell **9**, 402–411 (2011). 22056138 10.1016/j.stem.2011.10.004 PMC3212030

[36] K.-Z. Lee , Enterocyte purge and rapid recovery is a resilience reaction of the gut epithelium to pore-forming toxin attack. Cell Host Microbe **20**, 716–730 (2016). 27889464 10.1016/j.chom.2016.10.010

[37] N. Vodovar , Drosophila host defense after oral infection by an entomopathogenic Pseudomonas species. Proc. Natl. Acad. Sci. U.S.A. **102**, 11414–11419 (2005). 16061818 10.1073/pnas.0502240102 PMC1183552

[38] N. Buchon, N. A. Broderick, B. Lemaitre, Gut homeostasis in a microbial world: Insights from Drosophila melanogaster. Nat. Rev. Microbiol. **11**, 615–626 (2013). 23893105 10.1038/nrmicro3074

[39] O. Opota , Monalysin, a novel ß-pore-forming toxin from the Drosophila pathogen Pseudomonas entomophila, contributes to host intestinal damage and lethality. PLoS Pathog. **7**, e1002259 (2011). 21980286 10.1371/journal.ppat.1002259 PMC3182943

[40] P. Liehl, M. Blight, N. Vodovar, F. Boccard, B. Lemaitre, Prevalence of local immune response against oral infection in a Drosophila/Pseudomonas infection model. PLoS Pathog. **2**, e56 (2006). 16789834 10.1371/journal.ppat.0020056 PMC1475658

[41] M. S. Bou Sleiman , Genetic, molecular and physiological basis of variation in Drosophila gut immunocompetence. Nat. Commun. **6**, 7829 (2015). 26213329 10.1038/ncomms8829 PMC4525169

[42] B. Hudry, S. Khadayate, I. Miguel-Aliaga, The sexual identity of adult intestinal stem cells controls organ size and plasticity. Nature **530**, 344–348 (2016). 26887495 10.1038/nature16953 PMC4800002

[43] B. Hudry , Sex differences in intestinal carbohydrate metabolism promote food intake and sperm maturation. Cell **178**, 901–918.e16 (2019). 31398343 10.1016/j.cell.2019.07.029 PMC6700282

[44] T. F. C. Mackay , The Drosophila melanogaster genetic reference panel. Nature **482**, 173–178 (2012). 22318601 10.1038/nature10811 PMC3683990

[45] K. Asahina, Sex differences in Drosophila behavior: Qualitative and quantitative dimorphism. Curr. Opin. Physiol. **6**, 35–45 (2018). 30386833 10.1016/j.cophys.2018.04.004 PMC6205217

[46] S. Peedikayil-Kurien, H. Setty, M. Oren-Suissa, Environmental experiences shape sexually dimorphic neuronal circuits and behaviour. FEBS J. **291**, 1080–1101 (2024). 36582142 10.1111/febs.16714

[47] R. A. Schwenke, B. P. Lazzaro, M. F. Wolfner, Reproduction-immunity trade-offs in insects. Annu. Rev. Entomol. **61**, 239–256 (2016). 26667271 10.1146/annurev-ento-010715-023924 PMC5231921

[48] Y. Yu, I. Iatsenko, Drosophila symbionts in infection: When a friend becomes an enemy. Infect. Immun. **93**, e00511-24 (2025). 40172541 10.1128/iai.00511-24 PMC12070757

[49] D. Duneau , Stochastic variation in the initial phase of bacterial infection predicts the probability of survival in *D. melanogaster*. Elife **6**, e28298 (2017). 29022878 10.7554/eLife.28298 PMC5703640

[50] M. M. Ceder , CG4928 is vital for renal function in fruit flies and membrane potential in cells: A first in-depth characterization of the putative solute carrier UNC93A. Front. Cell Dev. Biol. **8**, 580291 (2020). 33163493 10.3389/fcell.2020.580291 PMC7591606

[51] D. P. Leader, S. A. Krause, A. Pandit, S. A. Davies, J. A. T. Dow, FlyAtlas 2: A new version of the Drosophila melanogaster expression atlas with RNA-Seq, miRNA-Seq and sex-specific data. Nucleic Acids Res. **46**, D809–D815 (2018). 29069479 10.1093/nar/gkx976 PMC5753349

[52] D. Currò, K+ channels as potential targets for the treatment of gastrointestinal motor disorders. Eur. J. Pharmacol. **733**, 97–101 (2014). 24726846 10.1016/j.ejphar.2014.03.049

[53] D. Cosme, M. M. Estevinho, F. Rieder, F. Magro, Potassium channels in intestinal epithelial cells and their pharmacological modulation: A systematic review. Am. J. Physiol. Cell Physiol. **320**, C520–C546 (2021). 33326312 10.1152/ajpcell.00393.2020 PMC8424539

[54] A. Gomes, E. Fernandes, J. L. F. C. Lima, Fluorescence probes used for detection of reactive oxygen species. J. Biochem. Biophys. Methods **65**, 45–80 (2005). 16297980 10.1016/j.jbbm.2005.10.003

[55] Y. Samuni, S. Goldstein, O. M. Dean, M. Berk, The chemistry and biological activities of N-acetylcysteine. Biochim. Biophys. Acta **1830**, 4117–4129 (2013). 23618697 10.1016/j.bbagen.2013.04.016

[56] I. Iatsenko, J.-P. Boquete, B. Lemaitre, Microbiota-derived lactate activates production of reactive oxygen species by the intestinal NADPH oxidase nox and shortens Drosophila lifespan. Immunity **49**, 929–942.e5 (2018). 30446385 10.1016/j.immuni.2018.09.017

[57] M. Kazek , Glucose and trehalose metabolism through the cyclic pentose phosphate pathway shapes pathogen resistance and host protection in Drosophila. PLoS Biol. **22**, e3002299 (2024). 38713712 10.1371/journal.pbio.3002299 PMC11101078

[58] T. TeSlaa, M. Ralser, J. Fan, J. D. Rabinowitz, The pentose phosphate pathway in health and disease. Nat. Metab. **5**, 1275–1289 (2023). 37612403 10.1038/s42255-023-00863-2 PMC11251397

[59] M. B. Hughes, J. C. Lucchesi, Genetic rescue of a lethal “Null” activity allele of 6-phosphogluconate dehydrogenase in drosophila melanogaster. *Science* **196**, 1114–1115 (1977). 10.1126/science.404711 404711

[60] H. B. Xie , The NADPH metabolic network regulates human αB-crystallin cardiomyopathy and reductive stress in *Drosophila melanogaster*. PLoS Genet. **9**, e1003544 (2013). 23818860 10.1371/journal.pgen.1003544 PMC3688542

[61] A. Sittka, V. Pfeiffer, K. Tedin, J. Vogel, The RNA chaperone Hfq is essential for the virulence of Salmonella typhimurium. Mol. Microbiol. **63**, 193–217 (2007). 17163975 10.1111/j.1365-2958.2006.05489.x PMC1810395

[62] C. Pichon, B. Felden, Small RNA genes expressed from Staphylococcus aureus genomic and pathogenicity islands with specific expression among pathogenic strains. Proc. Natl. Acad. Sci. U.S.A. **102**, 14249–14254 (2005). 16183745 10.1073/pnas.0503838102 PMC1242290

[63] E. Bode , Promoter activation in Δhfq mutants as an efficient tool for specialized metabolite production enabling direct bioactivity testing. Angew. Chem. Int. Ed. Engl. **58**, 18957–18963 (2019). 31693786 10.1002/anie.201910563 PMC6972681

[64] A. F. Seixas, A. F. Q. Silva, J. P. Sousa, C. M. Arraiano, J. M. Andrade, The RNA chaperone Hfq is a novel regulator of catalase expression and hydrogen peroxide-induced oxidative stress response in Listeria monocytogenes EGD-e. Free Rad. Biol. Med. **227**, 103–116 (2025). 39608557 10.1016/j.freeradbiomed.2024.11.038

[65] M. G. Moule, D. M. Monack, D. S. Schneider, Reciprocal analysis of francisella novicida infections of a Drosophila melanogaster model reveal host-pathogen conflicts mediated by reactive oxygen and imd-regulated innate immune response. PLoS Pathog. **6**, e1001065 (2010). 20865166 10.1371/journal.ppat.1001065 PMC2928790

[66] M. Shaka, A. Arias-Rojas, A. Hrdina, D. Frahm, I. Iatsenko, Lipopolysaccharide-mediated resistance to host antimicrobial peptides and hemocyte-derived reactive-oxygen species are the major Providencia alcalifaciens virulence factors in Drosophila melanogaster. PLoS Pathog. **18**, e1010825 (2022). 36084158 10.1371/journal.ppat.1010825 PMC9491580

[67] A. Arias-Rojas, D. Frahm, R. Hurwitz, V. Brinkmann, I. Iatsenko, Resistance to host antimicrobial peptides mediates resilience of gut commensals during infection and aging in Drosophila. Proc. Natl. Acad. Sci. U.S.A. **120**, e2305649120 (2023). 37639605 10.1073/pnas.2305649120 PMC10483595

[68] A. Arias-Rojas , MprF-mediated immune evasion is necessary for Lactiplantibacillus plantarum resilience in the Drosophila gut during inflammation. PLoS Pathog. **20**, e1012462 (2024). 39159259 10.1371/journal.ppat.1012462 PMC11361745

[69] W. I. Khan , Critical role for signal transducer and activator of transcription factor 6 in mediating intestinal muscle hypercontractility and worm expulsion in Trichinella spiralis-infected mice. Infect. Immun. **69**, 838–844 (2001). 11159976 10.1128/IAI.69.2.838-844.2001 PMC97960

[70] L. Ye , Enteroendocrine cells sense bacterial tryptophan catabolites to activate enteric and vagal neuronal pathways. Cell Host Microbe **29**, 179–196.e9 (2021). 33352109 10.1016/j.chom.2020.11.011 PMC7997396

[71] X. Zhao, J. Karpac, Glutamate metabolism directs energetic trade-offs to shape host-pathogen susceptibility in Drosophila. Cell Metab. **33**, 2428–2444.e8 (2021). 34710355 10.1016/j.cmet.2021.10.003 PMC9153082

[72] H. Westlake , Reproducibility of scientific claims in Drosophila immunity: A retrospective analysis of 400 publications. Elife **15**, RP108404 (2026).

[73] J. R. Feliciano, A. M. Grilo, S. I. Guerreiro, S. A. Sousa, J. H. Leitão, Pore. Fut. Microbiol. **11**, 137–151 (2016). 10.2217/fmb.15.128 26685037

[74] E. Sonnleitner , Reduced virulence of a hfq mutant of Pseudomonas aeruginosa O1. Microb. Pathog. **35**, 217–228 (2003). 14521880 10.1016/s0882-4010(03)00149-9

[75] M. Rubinić ., Proteomics data from “Sex differences in drosophila intestinal metabolism contribute to sexually dimorphic infection outcome and alter gut pathogen virulence.” ProteomeXchange Consortium. https://www.ebi.ac.uk/pride/archive/projects/PXD064190. Deposited 22 May 2025. 10.1073/pnas.2514992123 PMC13320730 42341050

[76] M. Rubinić ., RNA-seq data from “Sex differences in drosophila intestinal metabolism contribute to sexually dimorphic infection outcome and alter gut pathogen virulence.” NCBI BioProject. https://www.ncbi.nlm.nih.gov/sra/PRJNA1256345. Deposited 29 April 2025. 10.1073/pnas.2514992123 PMC13320730 42341050

