# Supplementary material for: Sex differences in Drosophila intestinal metabolism contribute to sexually dimorphic infection outcome and alter gut pathogen virulence
Source: Proc Natl Acad Sci U S A. 2026 Jun 24;123(26):e2514992123. doi: 10.1073/pnas.2514992123 (PMC13320730; doi:10.1073/pnas.2514992123)
Supplement: Supplementary file 1 — Appendix 01 (PDF) [file pnas.2514992123.sapp.pdf]

Supporting Information for

Sex Differences in *Drosophila* Intestinal Metabolism Contribute to Sexually Dimorphic Infection Outcome and Alter Gut Pathogen Virulence

Marko Rubinić[1, 2], Yi Yu[1], Aranzazu Arias-Rojas[1, 8], Kaisy A. Martinez[1, 9], Wioletta Klimek[1, 10], Dagmar Frahm[1], Volker Brinkmann[3], Nicole Paczia[4], Kathirvel Alagesan[5], David Duneau[6, 7], and Igor Iatsenko[1, *]

[1] Research group Genetics of host-microbe interactions, Max Planck Institute for Infection Biology, Charitéplatz 1, 10117 Berlin, Germany
[2] Humboldt-Universität zu Berlin, Faculty of Life Sciences, 10099 Berlin, Germany
[3] Microscopy Core Facility, Max Planck Institute for Infection Biology
[4] Core Facility for Metabolomics and Small Molecule Mass Spectrometry, Max Planck Institute for Terrestrial Microbiology, Marburg, Germany
[5] Max Planck Unit for the Science of Pathogens, Charitéplatz 1, 10117 Berlin, Germany
[6] Centre for Cardiovascular Science, Queen's Medical Research Institute, University of Edinburgh, Edinburgh, UK
[7] Center for Ecology, Evolution and Environmental Changes (cE3c) & Global Change and Sustainability Institute (CHANGE), Faculty of Sciences, University of Lisbon (FCUL), Lisbon, Portugal
[8] Present address: Broad Institute of MIT and Harvard, Cambridge, MA, USA
[9] Present address: University of Texas at Arlington, 701 S Nedderman Dr, Arlington, TX 76019
[10] Present address: Technische Universität Berlin, Straße des 17. Juni 135, 10623 Berlin

Corresponding author: Igor Iatsenko
Email: iatsenko@mpiib-berlin.mpg.de

This PDF file includes:

Supporting text
Figures S1 to S9
Legends for Datasets S1 to S9
Legends for Movies S1 to S4
SI References

Other supporting materials for this manuscript include the following:

Datasets S1 to S9
Movies S1 to S4

Supporting Information text

Supporting discussion

AMP-independent role of Imd pathway in host defense

Notably, females are more resistant than males to *Ecc15* intestinal infection which was attributed to the increased ability of females to repair their epithelium via ISC proliferation (1). Given that *P. entomophila* prevents intestinal repair, this mechanism is no longer advantageous to females during *P. entomophila* infection. Inhibition of AMP production via translation blockage in wild-type flies, raises a question of why Imd pathway mutants not able to produce AMPs are more susceptible to *P. entomophila* infection. This phenotype suggests that the Imd pathway has a likely, but as yet undefined, AMP-independent role in host protection against *P. entomophila* infection (2).

The cause of sexual dimorphism in PPP

Our results suggest that sex differences in PPP and antioxidant capacity prior to infection mediate observed differences in survival. But what is the cause of sexual dimorphism in carbohydrate metabolism? Hudry et al (3) identified that interorgan communication between male gonad and gut determines male bias in the carbohydrate metabolism. Specifically, male gonad activates JAK-STAT signaling in the enterocytes of adjacent gut region consequently inducing the expression carbohydrate metabolism genes. Hence, sexual dimorphism in survival to intestinal infection likely stems from sex differences in gut metabolism driven by JAK-STAT pathway-mediated testis-gut interorgan communication.

Supporting methods

Arrive protocol

The ARRIVE 2.0 guidelines were followed in planning, conducting and reporting *in vivo* experiments (4).

Drosophila husbandry

Drosophila stocks were raised in a light:dark cycle (12 h:12 h) at 25°C, on a standard cornmeal/agar medium (6.2 g agar, 58.8 g cornmeal, 58.8 g inactivated dried yeast, 26.5 ml of a 10% solution of methyl-paraben in 85% ethanol, 60 ml fruit juice, 4.8 ml 99% propionic acid for 1 L). For maintaining flies, flies were transferred to fresh vials every 2-3 days, and fly density was kept to a maximum of 15 flies per vial. At day 2 after eclosion, all emerged adults were transferred to new vials where they were left to mate for 2-3 days. Flies were then sorted using CO_2 in vials with 20 flies per sex the evening before experiment, unless co-housing the sexes was the aim of the experiment. Flies were then kept at 29°C, unless otherwise stated. Axenic flies were generated by egg bleaching as previously described (5, 6).

Drosophila strains and crosses

Drosophila strains used in this study are listed in Table S7. For preparing crosses, five males carrying UAS transgenes were placed together with five virgin females carrying GAL4 driver and transferred to fresh vials every 2-3 days at 18°C. At day 2 after eclosion, all emerged adults were transferred to vials and kept for 2 days at 18°C to allow mating, and then transferred to 29°C where they were kept for at least 2 days before sorting for experiments. All fly stocks were confirmed to be free of of known viruses and parasites.

Oral infection and survival assay

Unless otherwise indicated, the previously established protocol (Figure 1A) was used for the oral infection of flies (7). *Pseudomonas entomophila* L48 strain was obtained from Bruno Lemaitre's lab. Previously published *P. entomophila* Δ*hfq* mutant (8) was kindly provided by Dr. Edna Bode. *P. entomophila* strains utilized in this study were grown directly from frozen 50% glycerol stocks. Briefly, 10 µL of the stocks was incubated in 20 mL of LB medium in a 50 mL flask overnight (~16 h) at 29°C with shaking at 175 rpm. The following day, the overnight culture was diluted 1:16 in 150 mL of LB medium and incubated in 500 mL flasks under the same conditions for a minimum of an additional 24 hours. Before the infection experiment, the cultures were centrifuged at 3500 g and 4°C for 15 minutes, and their concentration was estimated to the optical density (OD) of 200 using PBS. The bacterial suspension

was then mixed 1:1 with a 5% sucrose solution prior to the infection experiment, and 150 μL of this mixture was pipetted into vials containing standard food and filter papers (referred to as infection vials). Before transferring mated flies (20 per vial) to the infection vials, the flies were kept in empty vials for 2 hours at 29°C (dry starvation). Flies were always infected at 6 Zeitgeber Time (the middle of the 12-hour light cycle) and were kept in the infection vials for 24 hours (unless 0.5 h protocol was used where flies were kept in the infection vials for 0.5 hours) before being transferred to conventional fly food vials. All infections were performed at 29°C. Flies were counted at the following time points: 0.5 h, 18 h, 24 h, 30 h, 42 h, 48 h, 54 h, 66 h, 72 h, and 78 h. Flies counted as 'dead' at the 0.5 h mark were excluded from the survival analysis due to mortality caused by drowning. All experiments were performed at least twice on different days with at least two vials per day, each containing around 20 flies.

Imaging and image analysis
At 16 hours after infection or sucrose-control treatment, guts were dissected and immediately fixed in 1× PBS containing 4% paraformaldehyde for 30 min at room temperature. Samples were then rinsed three times with 1× PBS and incubated for 30 min in 500 μl blocking buffer (1% bovine serum albumin, 0.05% sodium azide, and 0.05% Tween-20 dissolved in PBS). 1 μl of Hoechst 33342 (10 mg/ml; final dilution 1:500) was then added to 500 μl PBS, and samples were additionally incubated for 1 h at room temperature in the dark. Guts were then mounted in Mowiol 4% (Sigma-Aldrich). Images were acquired using a Leica Thunder microscope. Z-stacks of 80 - 100 μm were collected using a z-step size of 10 μm. No quantitative image analysis was performed - images were inspected only qualitatively to identify gut regions with missing nuclei or the presence of cytoplasmic DNA.

GWAS (Survival, Analysis, and Validation)

- **(a) Survival**

Survival of DGRP lines was performed as above with addition of 'stricter' control of mating density (vials where 12 males and 12 females were mated for 48 hours before sorting the flies for experiments) and additionally, counting of the dead flies was performed every 3 h instead of every 6 h during 12 h light cycle.

- **(b) Analysis**

We performed three GWA analyses for each data set: males' dataset (HR of male DGRP line relative to the reference line male w1118 iso), females dataset HR of female DGRP line relative to the reference line female w1118 iso), and sexual dimorphism dataset. (HR of female relative to the male of the DGRP line). HR was estimated using a Cox proportional hazards mode (coxph(Surv(Time_to_death_in_day,Censor) ~ DGRP_lines + DGRP_lines:Sex + strata(Day_of_exposure)). This allowed to calculate the hazard ratios (HR) for each genotype relative to *w1118* iso line, which was present in every experiment. To identify SNPs associated with variation in HR, we performed a GWAS using a linear model (lm(HR ~ SNP, weights = 1/(SE^2))). Weighting by the inverse of the variance (1/SE²) allows to give less weight to genotypes with badly estimated HR in the linear model.

- **(c) Functional validation**

To obtain an unbiased approach in gene selection, genes were selected by three authors (D.D., I.I., M.R.). We used multiple criteria such as the previously described function, the functional impact of the mutation as predicted by Ensembl Variant Effect Predictor (i.e. missense, UTR, within gene or intron/splice region), our transcriptomic and proteomics data, and the size of the effects observed on the DGRP lines (i.e. log (HR)). Validation of genes was performed by performing three independent survival assays (predetermined, crosses preparation on separate days with different flies) on crosses where selected candidate genes was downregulated by RNAi in using enterocyte specific GAL4 driver. For preparation of crosses (described above), all GAL4 virgins were collected over a period of five days and then on the day of cross preparation mixed altogether and randomly separated into vials with appropriate males. Survival assays were done in parallel (all genes) and counting of dead flies was performed every 3 hours during 12 h light cycle.

Food intake quantification

Quantifying the amount of ingested food was done by including blue dye (Food Blue No. 1, TCI) in infection mixture as described previously (9). *P. entomophila* was mixed in a 1:1 ratio with a 5% sucrose solution containing 1% blue dye. Feeding was interrupted after 0.5 h, and five flies were immediately transferred to pre-weighed tubes containing glass beads and 500 µL of 1x PBS with 0.1% Triton X-100 (PBST). The tubes with the flies were then weighed, allowing for the calculation of the flies' weight. The samples were then homogenized using a Precellys homogenizer (three cycles of 30 seconds at 7200 rpm) and centrifuged twice (2 mins, maximum speed). 250 µL of supernatant was then transferred to cuvette containing 750 µL of water. Blue dye levels were quantified by measuring absorbance at 630 nm using Ultrospec 2100 pro UV/Vis Spectrometer (Amersham Biosciences). Flies fed with *P. entomophila* mixed 1:1 with 5% sucrose without blue dye were used as a background control. Values were converted to the amount of blue dye (in ng) using a calibration curve obtained from the serial dilutions of a blue dye in PBST and then normalized per weight (in mg) of flies.

Gut transcriptome comparison (RNA Extraction, RNAseq and GO analysis)
Total RNA was extracted from 30 guts per sample using TRIzol reagent (Invitrogen). RNA quantity and quality were measured using a NanoDrop 2000 (Thermo Scientific). For each condition, RNAs were prepared from three biological replicates on different days. RNA-seq was carried out by Novogene (Cambridge, UK) following standard Illumina protocols. Briefly, RNA quantity, integrity and purity were determined using the Agilent 5400 Fragment Analyzer System (Agilent Technologies). mRNAs were purified from total RNA using poly-T oligonucleotide-attached magnetic beads. After fragmentation, the first-strand cDNA was synthesized using random hexamer primers. Then the second-strand cDNA was synthesized using dUTP, instead of dTTP. The directional library was ready after end repair, A-tailing, adapter ligation, size selection, USER enzyme digestion, amplification and purification. The libraries were quantified with Qubit and checked with bioanalyser for size distribution. Quantified libraries were pooled and sequenced on the Illumina NovaSeq 6000 platform (2 × 150 bp) and generated about 6 Gb of raw data per sample.
We characterized male and female transcriptional response to infection (infected guts compared to sucrose-fed (control) guts of matching sex) and difference between male and female transcriptome under non-infected conditions (male sucrose-fed guts compared to female sucrose-fed guts). Differential expression analysis was done using DESeq2(10). Significance cut-offs were padj < 0.1, $|log_2FC| > 1.5$. Scatter plot with gene categories (genes where the response (infected vs. sucrose-fed (control)) was upregulated or downregulated in both sexes, 'female biased' - genes where response (either up or down) was significant only in female flies, and not male flies, 'male biased' - genes where response (either up or down) was significant only in male flies, and not female flies) was used to visualize comparison between male and female transcriptional response to infection. KEGG enrichment analysis for gene group lists (comparison of non-infected conditions) was done using enrichKEGG function from the clusterProfiler package. The p-value cutoff was 0.05. The R package ggplot2was used for data visualization.
All raw RNA sequencing data files are available from the SRA database (accession number PRJNA1256345) and can be accessed at https://www.ncbi.nlm.nih.gov/sra/PRJNA1256345 on the day of publication.

Protein concentration measurement
Protein concentrations were measured by the Pierce BCA Protein Assay Kit (Thermo Fisher Scientific Pierce™ BCA Protein Assay Kit Catalog Numbers 23225 and 23227) according to the manufacturer's protocol. Briefly, 10 µL of samples were incubated with 200 µL of working reagent and incubated for 30 min at 37°C. Absorbance measurements (in duplicates) were performed at 562 nm using a plate reader Infinite M Plex Microplate Reader (Tecan). For incompatible samples, Pierce™ 660 nm Protein Assay Reagent was used. Briefly, 10 µL of samples were incubated with 150 µL of the working reagent, mixed for 1 minute, incubated at room temperature (RT) for 5 minutes. Absorbance measurements (in duplicates) were performed at 660 nm using a plate reader Infinite M Plex Microplate Reader (Tecan).

Gut Proteome Comparison
At the indicated timepoints, 30 guts were dissected in 150 µl of protein extraction buffer (100 mM Tris-Cl pH 8, 2% SDS, protease inhibitor) on ice. Following incubation (95°C for 2 minutes) and homogenization (6000 rpm for 30 seconds, Precellys homogenizer), samples were centrifuges (max

speed, 10 min, 4°C). Supernatant was collected into Eppendorf tubes. Protein measurement was performed as described above (Pierce BCA Protein Assay Kit). Samples were stored at -80°C before being analyzed by High Throughput Mass Spectrometry Core Facility (Charité).

(a) Sample preparation (SP3)

Gut protein extracts were processed using the SP3 protocol as previously described with one-step reduction and alkylation (11).
Briefly, 16.6 µl of reduction and alkylation buffer (40 mM Tris(2-carboxyethyl)phosphine, 160 mM Chloroacetamide, 200 mM Ammonium bicarbonate, 4% SDS) was added, samples were incubated at 95 °C for 5 min and cooled to room temperature. To bind the proteins, 250 µg of paramagnetic beads (1:1 ratio hydrophilic/hydrophobic) were used, and the proteins were precipitated by adding 50% Acetonitrile. Samples were washed twice with 80% EtOH and once with 100% Acetonitrile. 35µl of 100 mM Ammonium bicarbonate and Trypsin/LysC (0.1 µg/µl stock solution protein: enzyme ratio of 1:50 (w/w)) were added and the lysates were incubated at 37°C with shaking overnight. The reaction was stopped by adding formic acid to a final concentration of 0.1%. Peptide concentration was determined using Pierce Quantitative Fluorometric Peptide Assay Kit, and peptide mixtures were analyzed by LC-MS/MS without further conditioning or clean-up.

(b) Proteome analysis by DIA LC-MS

Peptide separation was accomplished in a data independent acquisition (DIA) mode for a 63-minute in water to acetonitrile active gradient on an Ultimate 3000 RSLCnano HPLC coupled to a Q-Exactive Plus mass spectrometer (both ThermoFisher Scientific). 1 µg digested peptides were trapped on a column (PepMap C18, 5 mm x 300 µm x 5 µm, 100 Å, Thermo Fisher Scientific) with buffer containing 2:98 (v/v) acetonitrile/water in 0.1% (v/v) trifluoroacetic acid, flow rate of 20 µl/min for 3 min. The peptide mixture was separated on a C18 column (Acclaim PepMap C18, 2 µm; 100 Å; 75µm, Thermo Fisher Scientific) using a linear gradient from 5% to 28% buffer B over 63 minutes, followed by an increase to 95% buffer B in 2 minutes, and a 5-minute wash with 95% buffer B before a 20-minute equilibration with initial conditions. Buffer B is made of 80% acetonitrile and 0.1% formic acid mixed with LC-MS-grade water. Total acquisition time was 100 min. The Orbitrap worked in centroid mode including a duty cycle consisted of one MS1 scan at 70,000 resolution power with maximum injection time 300 ms and 3e6 AGC target followed by 40 variable MS2 scans using an 0.5 da overlapping window pattern. The acquisition started with window length of 25 MS2 scans at 12.5 da; followed by 7 windows with 25 da, then the last 8 windows were set to 62.5 da. The resolution of precursor MS spectra (m/z 378-1370) was set to 17,500 after accumulating ions for 110 ms to achieve a target value of 3e6. Mass spectrometric settings were set to: spray voltage, 2.0 kV; no sheath and auxiliary gas flow; heated capillary temperature, 275 °C; normalized HCD collision energy 27%. Additionally, the background ions m/z 391.2843 and 445.1200 acted as lock mass.

(c) Data analysis

Raw data were processed using DIA-NN 1.8.1 (12) with MS2 and MS1 mass accuracies set to 20 and 10 ppm, respectively. The output was filtered at 1% FDR on peptide level. Quantification strategy was "Robust LC with high precision". A spectral library free search with activated match between runs (MBR) was used (13).
We characterized male and female proteomic response to infection (infected guts compared to sucrose-fed (control) guts of matching sex) and difference between male and female proteome under non-infected conditions (male sucrose-fed guts compared to female sucrose-fed guts). The differential analysis of quantitative proteomics data was performed in Perseus v2.0.6.0. Significance cut-offs were padj < 0.05, |log2FC| > 1. Scatter plot with protein categories (proteins where the response (infected vs. sucrose-fed (control)) was upregulated or downregulated in both sexes, 'female biased' - proteins where response (either up or down) was significant only in female flies, and not male flies, 'male biased' - proteins where response (either up or down) was significant only in male flies, and not female flies) was used to visualize comparison between male and female proteomic response to infection. KEGG enrichment analysis for protein group lists (comparison of non-infected conditions) was done using

enrichKEGG function from the clusterProfiler package. The p-value cutoff was 0.05. The R packages ggplot2 was used for data visualization.
The mass spectrometry proteomics data have been deposited to the ProteomeXchange Consortium via the PRIDE partner repository with the dataset identifier PXD064190.

Bacterial load measurement
Bacteria loads were recorded by CFUs after infecting flies for 0.5 hours and then flipping them to conventional vials. Samples from pooled groups of five flies were collected at 0.5, 2, 4, 6, and 24 hours since initial exposure to *P. entomophila*. The flies were first surface sterilized in 70% ethanol for 1 minute, following three washes in sterile PBS for 30 sec, and then homogenized in 500 µL of sterile PBS for 30 seconds at 6000 rpm using a Precellys 24 instrument (Bertin Technologies, France). Serial 10-fold dilutions, ranging from 10^{-1} to 10^{-6}, were made and plated on LB plates using an automatic diluter and plater (easySpiral Dilute (Interscience, France)). After overnight incubation (proxy 18 h) at 30°C, colonies were counted with an automatic colony counter (Scan 1200 (Interscience, France)), along with its accompanying software. As a control, flies fed with sucrose were processed in parallel with infected flies. Since the control plates were negative for bacterial colonies, it was assumed that all colonies that grew at plates of infected flies are *P. entomophila*.

Measuring defecation in adult flies
To measure defecation, we prepared and infected flies with a suspension containing blue dye as described above (see: Food intake quantification). Flies were kept on blue suspension for 0.5 h and then transferred to the vial containing conventional non-colored food for an additional 1.5 hours (time points were chosen to reflect bacterial load experiment (see: Figure 3A) where we observed first reduction of bacteria in male flies). Defecation rate was measured by counting ‘defecation dots’ left dried on the inner wall of vials and normalized per number of flies in vial. Experiments were performed at least twice on different days, with a total of at least 10 independent vials per condition, each containing 10 or 20 flies.

Ex vivo quantification of gut contractions
At 1h post treatment (as described above), vials were transferred to room temperature next to the microscope. In a period of 1h to 2h post start of the experiment, vial per vial was briefly placed on ice before taking one fly for dissection. Upon awaking of the fly, guts were carefully dissected directly in Schneider’s medium, transferred onto microscope slide type with concave indentions (VWR, LOT 0180) that contained 100 µl Schneider’s medium (room temperature). Samples were covered with r coverslips (Roth, 24 × 24 mm, #1, Art. No. 875). Videos were acquired on a Leica Thunder microscope in dark field mode using a 5×/0.15 NA dry objective. A total recording time of 3 minutes where an image was taken every 200 ms.
Gut contractions were counted manually. Any visible contraction in any region of the gut was considered as a contraction readout. Quantification was done in the first minute of the recorded video. Each experiment was performed in at least three independent biological repeats, with a minimum of two guts imaged per repeat. Two female *w1118* iso sucrose treated samples were excluded from the analysis because they showed zero contractions, which has been previously reported as a potential artefact of the dissection procedure.

Chemical manipulations
For experiments where the effect of a compound on survival or defecation was tested, experiments were performed and described before, with the exception that either PBS (control) or *P. entomophila* were mixed 1:1 with sucrose-containing compounds. The final concentration (after 1:1 mix) were as follows: N-Methylmaleimide (NMM, Thermo Scientific, Cat# 127080050) was 1 mM, N-Acetyl-L-Cysteine (NAC, Sigma-Aldrich, Cat# A7250) was 20 mM, Methyl Viologen hydrate (98% (paraquat, (Thermo Scientific, Cat# 227320050)) was 20 mM).

ROS measurement
We used the dye 2′,7′- dichlorodihydrofluorescein diacetate (H_2DCF-DA, ≥97%, Invitrogen™, Cat# D399) which yields fluorescent dichlorofluorescein when oxidized as an indicator of general oxidative stress (14). At specified time points, 30 guts were dissected directly into 150 µL of PBS kept on ice. The samples were homogenized using glass beads (30 seconds, 6000 rpm, Precellys 24 instrument (Bertin

Technologies, France)). The entire homogenate was transferred to fresh Eppendorf tubes and centrifuged at maximum speed for 10 minutes at 4°C. 70 µL of the supernatant was transferred to a new tube and used for protein measurement, as described above (Pierce BCA Protein Assay Kit), and for measurement of general oxidative stress. 10 µL of gut homogenate was mixed with 190 µL of a 1x PBS solution containing 50 µM H_2DCF-DA and incubated for 30 minutes at 37°C in darkness. Fluorescence was measured using an excitation wavelength of 488 nm and an emission wavelength of 520 nm. Auto-oxidation of H_2DCF-DA served as a control. All samples were measured in duplicates and the levels of ROS were normalized as a percentage of relative DCF fluorescence units per microgram of protein in the sample.
To quantify gut H_2O_2 levels, we used the Fluorimetric Hydrogen Peroxide Assay Kit (Sigma-Aldrich, Cat# MAK165) according to the manufacturer's instructions. Samples were prepared as described for the H2DCF-DA assay, with the exception of dilution, where samples were diluted (1:10 for male samples and 1:20 for female samples) to a final volume of 50 µL. Fluorescence was measured at an excitation wavelength of 540 nm and an emission wavelength of 590 nm. All samples were measured in duplicates, corrected for dilution factors, and H_2O_2 levels were normalized to protein content (per µg protein).

Metabolites Comparison
Five whole flies per sample were used following the 6 h sucrose-feeding (control that was used for RNAseq and proteomics). To extract the metabolites, flies were homogenized using a Precellys homogenizer (6000 rpm, 30 seconds) in a mixture of 100 µL of 50% methanol and 150 µL of chloroform. Following centrifugation (10,000 g, 20 minutes, 4°C), 50 µL of the upper aqueous layer were transferred into HPLC/GC certified vials (Fisherbrand™) on ice. Protein concentration was measured as described above (Pierce™ 660 nm Protein Assay Reagent). Rest of the samples were kept at -80°C before sending to Core facility for metabolomics and small molecules mass spectrometry (Max Planck Institute for Terrestrial Microbiology, Marburg) for metabolomic analysis.
Quantitative metabolite determination was performed using a LC-MS/MS. The chromatographic separation was performed on an Agilent Infinity II 1290 HPLC system using a SeQuant ZIC-pHILIC column (150 × 2.1 mm, 5 µm particle size, peek coated, Merck) connected to a guard column of similar specificity (20 × 2.1 mm, 5 µm particle size, Phenomoenex) a constant flow rate of 0.1 ml/min with mobile phase A with mobile phase comprised of 10 mM ammonium acetate in water, pH 9, supplemented with medronic acid to a final concentration of 5 µM (A) and 10 mM ammonium acetate in 90:10 acetonitrile to water, pH 9, supplemented with medronic acid to a final concentration of 5 µM (B) at 40°C .
The injection volume was 1 µl. The mobile phase profile consisted of the following steps and linear gradients: 0 – 1 min constant at 75% B; 1 – 6 min from 75 to 40% B; 6 to 9 min constant at 40% B; 9 – 9.1 min from 40 to 75% B; 9.1 to 20 min constant at 75% B. An Agilent 6495 ion funnel mass spectrometer was used in positive and negative mode with an electrospray ionization source and the following conditions: ESI spray voltage 2000 V(-)/3500 V(+), nozzle voltage 1000 V, sheath gas 300° C at 20 l/min, nebulizer pressure 20 psig and drying gas 100°C at 11 l/min. Compounds were identified based on their mass transition and retention time compared to standards. Chromatograms were integrated using MassHunter software (Agilent, Santa Clara, CA, USA).
Mass transitions, collision energies, Cell accelerator voltages, and Dwell times have been optimized using chemically pure standards. The parameter settings of all targets are given in Table S8.

NADPH/NADP+ KIT
The NADP+/NADPH ratio was measured from whole fly lysates using the NADP+/NADPH Quantification Colorimetric Kit (Abcam, Cat# ab65349) according to the manufacturer's protocol. Colorimetric measurements were performed at 450 nm using a plate reader Infinite M Plex Microplate Reader (Tecan). Five whole flies per sample were used following the 6 h sucrose-feeding (control that was used for RNAseq and proteomics). Values were normalized per protein concentration measured as described above (Pierce™ 660 nm Protein Assay Reagent).

Minimal Inhibitory Concentration (MIC)
Overnight bacterial cultures were adjusted to OD 0.1, diluted 1:100, and pipetted (50 µl) into wells of 96 well plates prefilled with ranging dilutions of 50 µl of Polymyxin B (Fischer Scientific) (nine 1:1 dilutions from initial 100 µl/mL and 40 µl/mL solutions (final minimal concertation tested 0.0781 µl/mL Polymyxin)., paraquat (Methyl Viologen hydrate, 98%, thermo scientific) (nine 1:1 dilutions from initial 15 mg/mL and

6 mg/mL solutions (final minimal concertation tested 0.0117 μg/mL paraquat)) or H_2O_2 (Hydrogen Peroxide Solution, Sigma-Aldrich) (nine 1:1 dilutions from initial 3% and 1.2% solutions (final minimal concertation tested 0.002 % μg/mL H_2O_2). After overnight incubation, the bacterial growth was recorded to determine MIC value. OD600 was measured. Reads were performed in Infinite M Plex Microplate Reader (Tecan).

Quantification and statistical analysis
Statistical parameters and tests are shown in the figure legends and Table S9. No formal randomization method was used, but to reduce the potential bias, we varied the order of sorting flies, infection vials, sampling and well plate location of samples across different biological replicates. Additionally, control and treatment groups were processed in parallel, and sample sizes were balanced. Blinding was not implemented in this study. No data were excluded from analysis. Data analyses were performed using GraphPad Prism 10 software and R v4.3.2. Kinetics of survival data were shown using cumulative data, and survival analysis was carried with Cox proportional hazards model using Survival R package. Log Hazard ratio is represented to provide the estimate of difference in survival considering all the parameters, including day of the experiment. 95% confidence intervals are provided by the package Survival and are approximated by calculating 1.96 times the standard error. Data visualization was performed with the R packages ggplot2, dplyr, and tidyverse. Statistical significance was determined using either the unpaired Student's t-test or Mixed-effects model (REML) or one-way or two-way ANOVA, followed by post-hoc tests for multiple comparisons (indicated in the figure legends). Significance was set at $p < 0.05$, with data presented as mean ± SEM. Significance: *$p < 0.05$, **$p < 0.01$, ***$p < 0.001$, ****$p < 0.0001$.

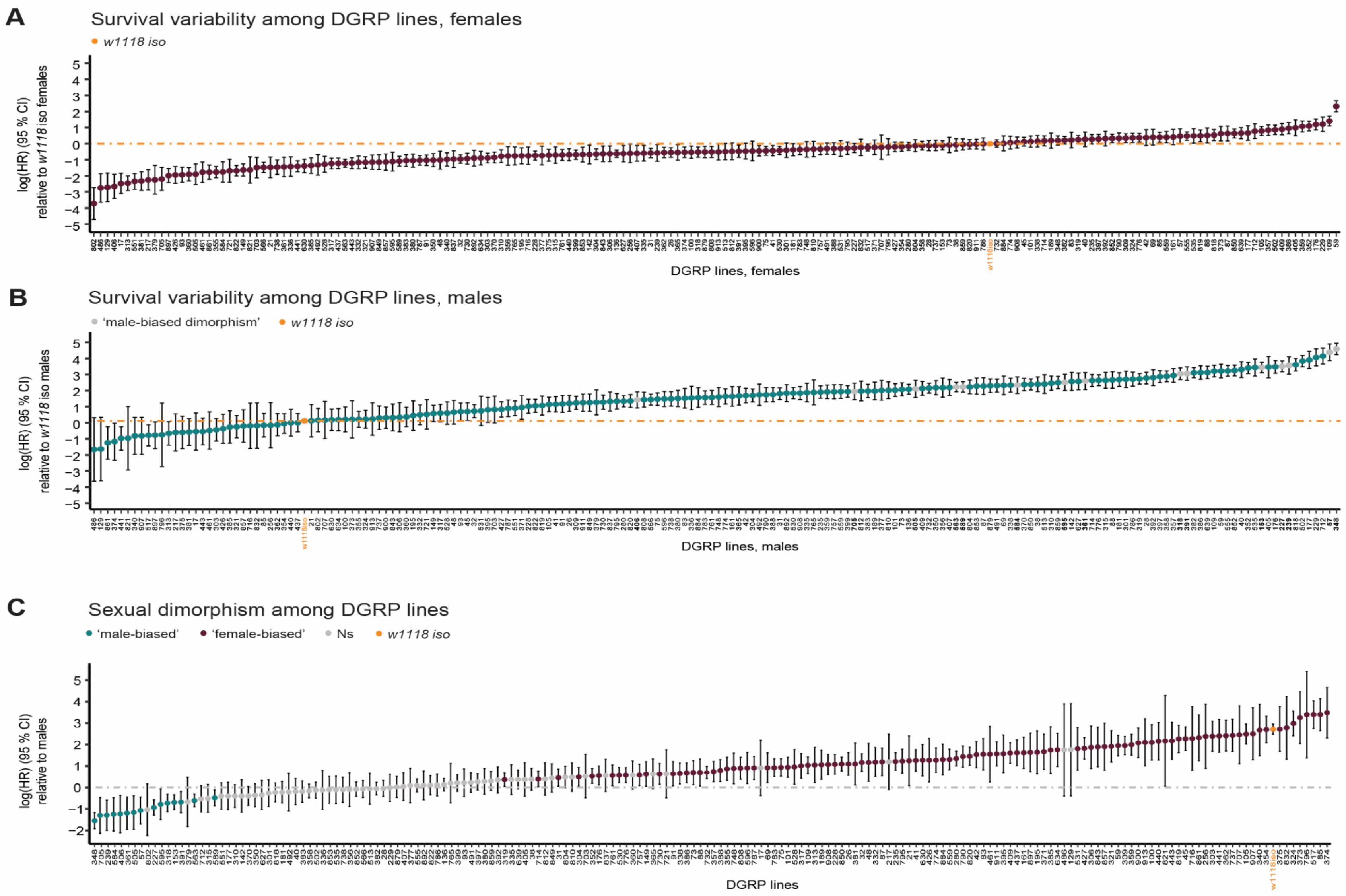

Fig. S1. Sexual dimorphism across DGRP lines.

(A and B) Variability in susceptibility to *P. entomophila* infection across each sex (**(A)** females and **(B)** males) of DGRP lines compared to the appropriate sex of the w^{1118} iso strain. (**C**) Variability in sexual dimorphism in susceptibility to *P. entomophila* infection across 183 DGRP lines (DGRP lines where female flies were more susceptible compared to male flies are highlighted in plum, those where male flies were more susceptible compared to female flies are highlighted in teal. The reference w^{1118} iso strain is in orange despite the female's bias)

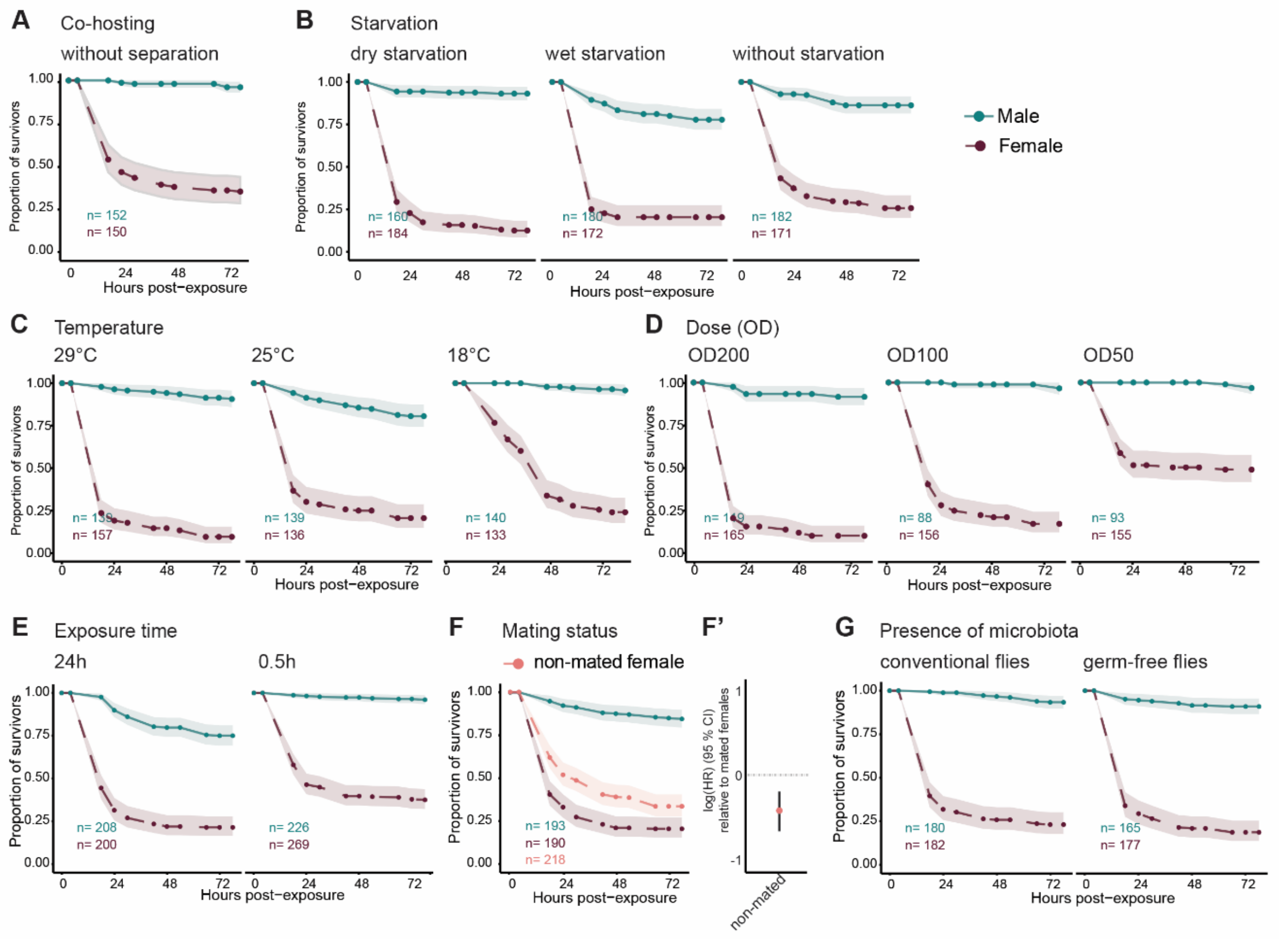

Fig. S2. Sexual dimorphism in survival to *P. entomophila* intestinal infection is common across experimental conditions.

(A – F, G) Survival curves with 95% confidence intervals (shaded area) of w^{1118} iso upon exposure to *P. entomophila* under different protocol conditions. Data is related to **Figure 1F – J, L, M.**

(F') Hazard ratios with 95% confidence intervals of non-mated w^{1118} iso females upon exposure to *P. entomophila* compared to mated w^{1118} iso females.

For detailed sample sizes and statistical analyses, see Table S9.

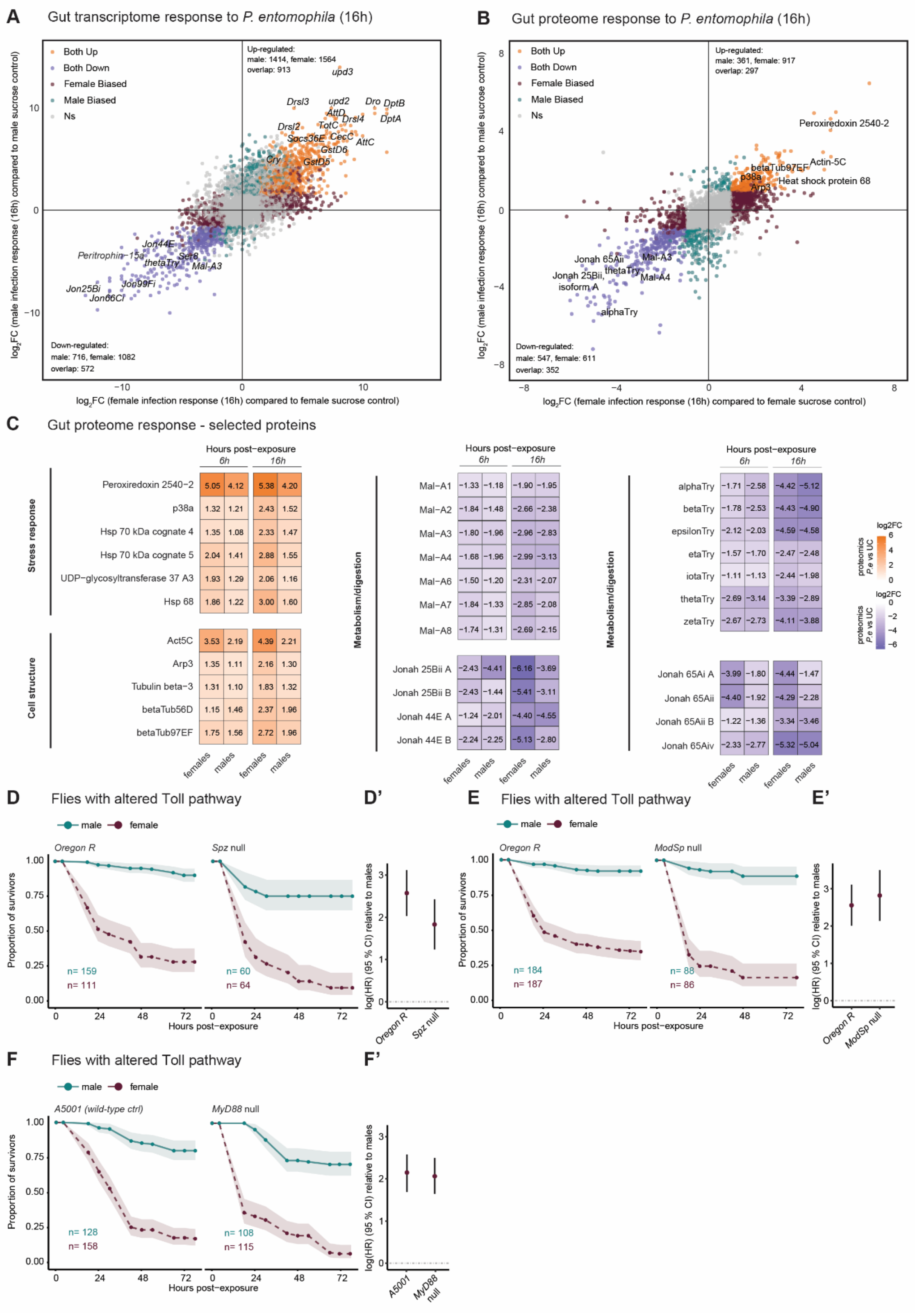
A
Gut transcriptome response to P. entomophila (16h)
Both Up
Both Down
Female Biased
Male Biased
Ns
Up-regulated:
male: 1414, female: 1564
overlap: 913
Down-regulated:
male: 716, female: 1082
overlap: 572
upd3
Drsl3
upd2
Dro
DptB
AttD
Drsl4
DptA
Drsl2
TotC
Socs36E
CecC
AttC
GstD6
Cry
GstD5
Jon44E
Peritrophin-15a
Ser8
thetaTry
Mal-A3
Jon99Fi
Jon25Bi
Jon66Ci
log2FC (male infection response (16h) compared to male sucrose control)
log2FC (female infection response (16h) compared to female sucrose control)
B
Gut proteome response to P. entomophila (16h)
Up-regulated:
male: 361, female: 917
overlap: 297
Down-regulated:
male: 547, female: 611
overlap: 352
Peroxiredoxin 2540-2
Actin-5C
betaTub97EF
p38a
Heat shock protein 68
Arp3
Jonah 65Aii
Mal-A3
thetaTry
Jonah 25Bii, isoform A
Mal-A4
alphaTry
C
Gut proteome response - selected proteins
Hours post-exposure
6h
16h
Stress response
Peroxiredoxin 2540-2 5.05 4.12 5.38 4.20
p38a 1.32 1.21 2.43 1.52
Hsp 70 kDa cognate 4 1.35 1.08 2.33 1.47
Hsp 70 kDa cognate 5 2.04 1.41 2.88 1.55
UDP-glycosyltransferase 37 A3 1.93 1.29 2.06 1.16
Hsp 68 1.86 1.22 3.00 1.60
Cell structure
Act5C 3.53 2.19 4.39 2.21
Arp3 1.35 1.11 2.16 1.30
Tubulin beta-3 1.31 1.10 1.83 1.32
betaTub56D 1.15 1.46 2.37 1.96
betaTub97EF 1.75 1.56 2.72 1.96
Metabolism/digestion
Mal-A1 -1.33 -1.18 -1.90 -1.95
Mal-A2 -1.84 -1.48 -2.66 -2.38
Mal-A3 -1.80 -1.96 -2.96 -2.83
Mal-A4 -1.68 -1.96 -2.99 -3.13
Mal-A6 -1.50 -1.20 -2.31 -2.07
Mal-A7 -1.84 -1.33 -2.85 -2.08
Mal-A8 -1.74 -1.31 -2.69 -2.15
Jonah 25Bii A -2.43 -4.41 -6.16 -3.69
Jonah 25Bii B -2.43 -1.44 -5.41 -3.11
Jonah 44E A -1.24 -2.01 -4.40 -4.55
Jonah 44E B -2.24 -2.25 -5.13 -2.80
alphaTry -1.71 -2.58 -4.42 -5.12
betaTry -1.78 -2.53 -4.43 -4.90
epsilonTry -2.12 -2.03 -4.59 -4.58
etaTry -1.57 -1.70 -2.47 -2.48
iotaTry -1.11 -1.13 -2.44 -1.98
thetaTry -2.69 -3.14 -3.39 -2.89
zetaTry -2.67 -2.73 -4.11 -3.88
Jonah 65Ai A -3.99 -1.80 -4.44 -1.47
Jonah 65Aii -4.40 -1.92 -4.29 -2.28
Jonah 65Aii B -1.22 -1.36 -3.34 -3.46
Jonah 65Aiv -2.33 -2.77 -5.32 -5.04
females
males
log2FC
proteomics P.e vs UC
D
Flies with altered Toll pathway
male
female
Oregon R
Spz null
n= 159
n= 111
n= 60
n= 64
Proportion of survivors
Hours post-exposure
D'
log(HR) (95 % CI) relative to males
E
Flies with altered Toll pathway
Oregon R
ModSp null
n= 184
n= 187
n= 88
n= 86
E'
F
Flies with altered Toll pathway
A5001 (wild-type ctrl)
MyD88 null
n= 128
n= 158
n= 108
n= 115
F'
A5001
MyD88 null

Fig. S3. Sexual dimorphism in survival to *P. entomophila* intestinal infection is not explained by the major immune pathways.

(A and B) Scatter plots representing log_2FC of **(A)** gene expression or **(B)** protein abundance in female (x) vs male (y) guts 16 h post-exposure to *P. entomophila* (infected guts compared to sucrose-fed (control) guts of matching sex). Significance cut-offs: **(A)** padj < 0.1, $|log_2FC| > 1.5$, and **(B)** padj < 0.05, $|log_2FC| > 1$. **(A)** (N = 3 independent samples, each with 30 pooled guts). **(B)** (N = 5 independent samples, each with 30 pooled guts).

(C) Heatmaps showing differences (log_2FC, infected guts compared to control guts) in the abundance of selected proteins of each sex at 6 h and 16 h post-exposure to *P. entomophila*.

(D and D') Survival curves with 95% confidence intervals (shaded area) and hazard ratios with 95% confidence intervals of Oregon R (control) and spz^{rm7} loss-of-function mutant upon exposure to *P. entomophila*.

(E and E') Survival curves with 95% confidence intervals (shaded area) and hazard ratios with 95% confidence intervals of Oregon R (control) and *ModSp* loss-of-function mutant upon exposure to *P. entomophila*.

(F and F') Survival curves with 95% confidence intervals (shaded area) and hazard ratios with 95% confidence intervals of A5001 (w1118 wt control) and *MyD88* loss-of-function mutant upon exposure to *P. entomophila*.

For detailed sample sizes and statistical analyses, see Table S9.

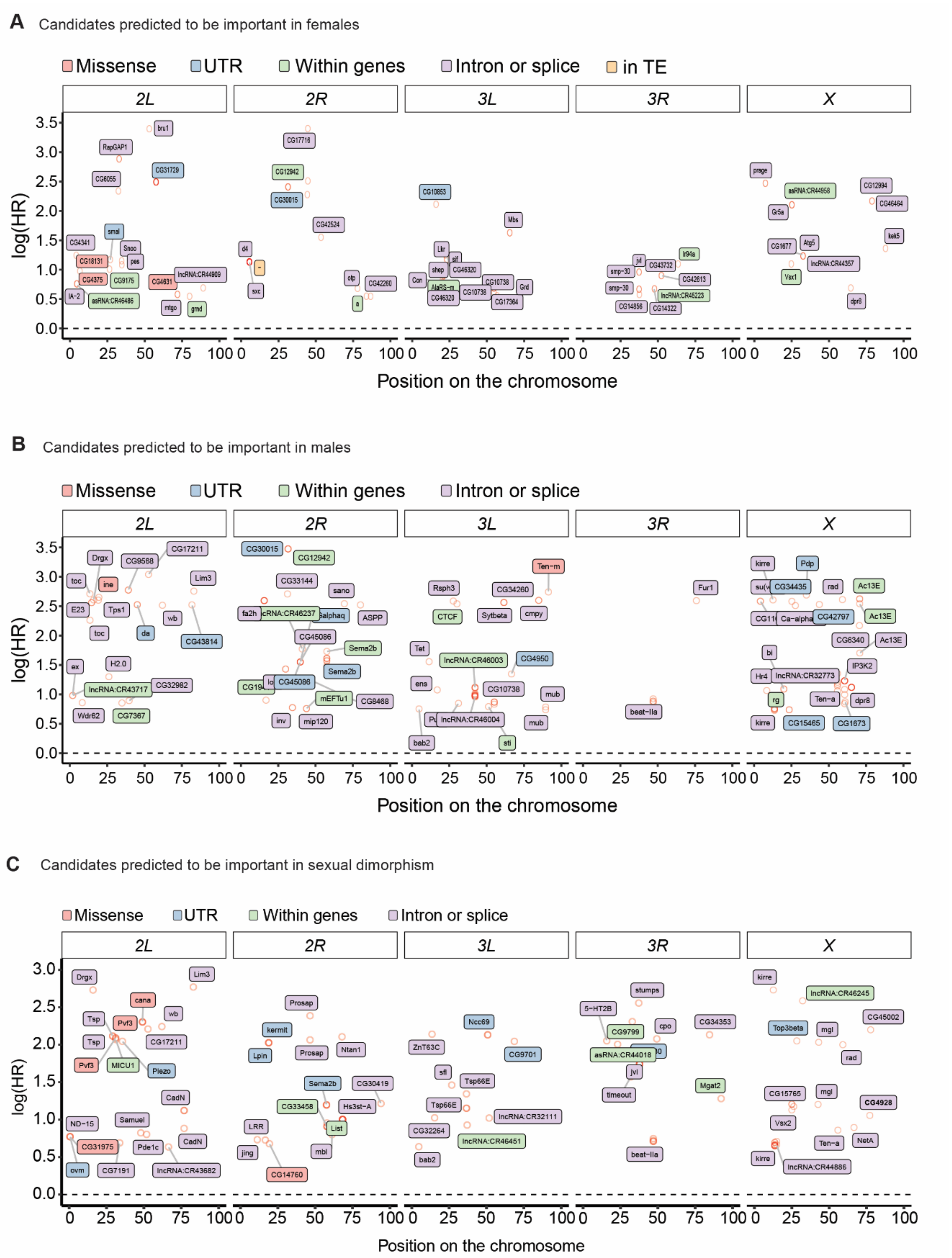

Fig. S4. GWAS-identified candidates.

(A and B) Candidate genes underlying natural variation in the susceptibility to *P. entomophila* gut infection among **(A)** female and **(B)** male flies.
(C) Candidate genes underlying natural variation in the sexual dimorphism to *P. entomophila* gut infection.

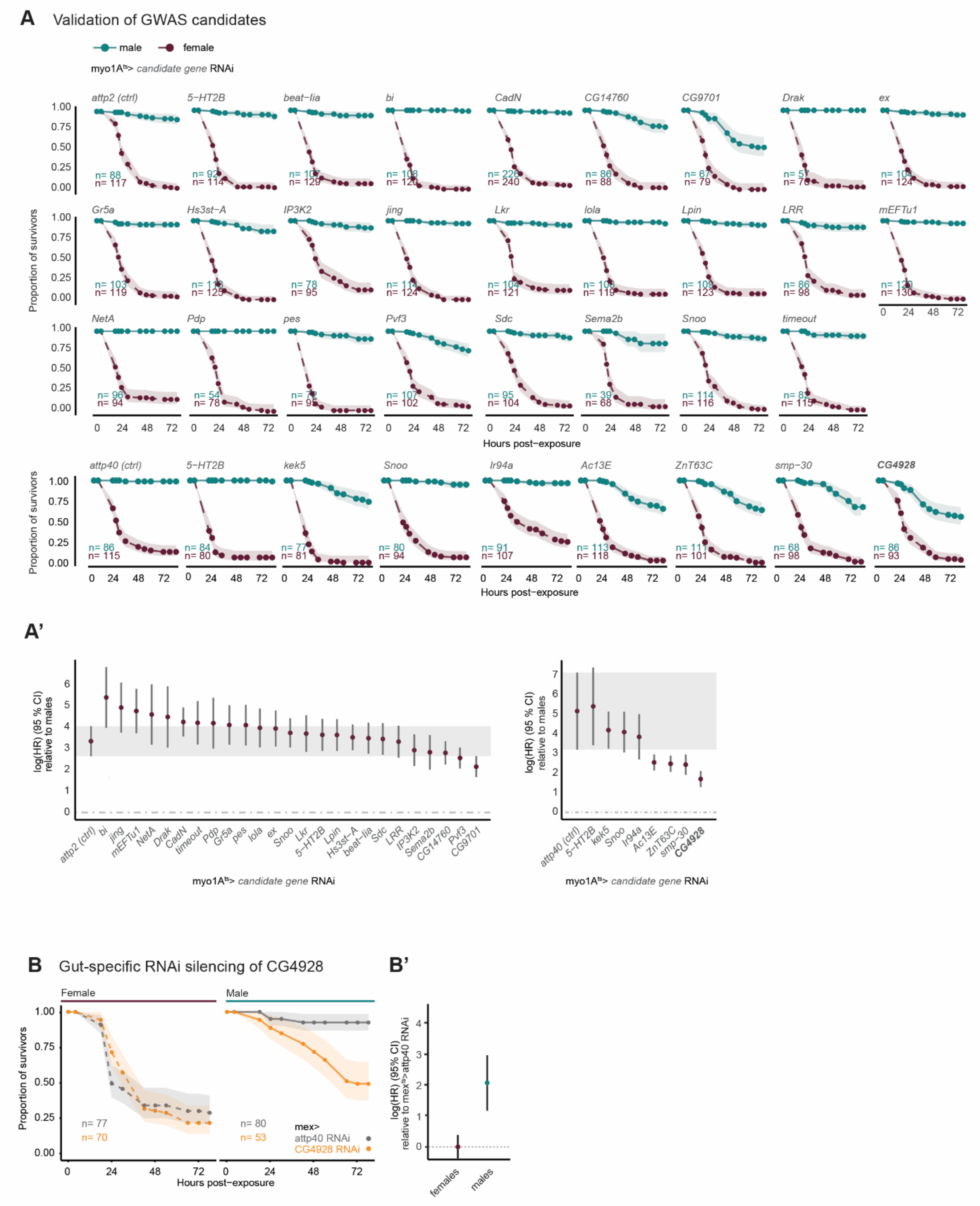

Fig. S5. Validation of GWAS-identified candidates revealed new mediators of male survival after infection.

(A and A') Survival curves with 95% confidence intervals (shaded area) and hazard ratios with 95% confidence intervals of *myo1A*^{ts}*>* crossed with appropriate control (*attp2* RNAi or *attp40* RNAi) and RNAi line for GWAS gene candidates upon exposure to *P. entomophila*.

(B and B') Survival curves with 95% confidence intervals (shaded area) and hazard ratios with 95% confidence intervals of *mex>attp40* RNAi (control) and *mex>CG4928* RNAi upon exposure to *P. entomophila*.

For detailed sample sizes and statistical analyses, see Table S9.

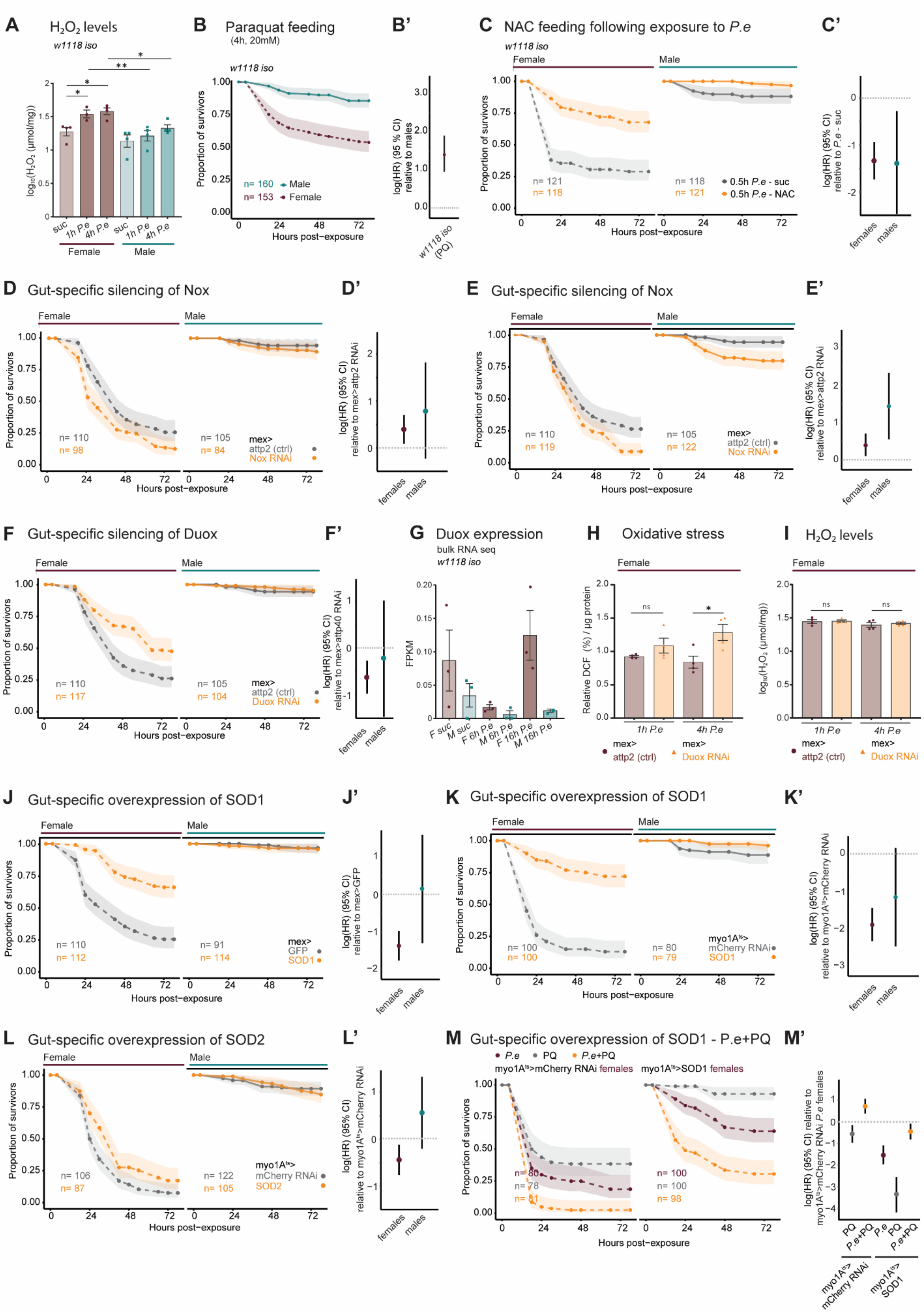
A H2O2 levels
w1118 iso
log10(H2O2 (µmol/mg))
suc
1h P.e
4h P.e
Female
Male
B Paraquat feeding
(4h, 20mM)
w1118 iso
Proportion of survivors
n= 160 Male
n= 153 Female
Hours post-exposure
B'
log(HR) (95 % CI) relative to males
w1118 iso (PQ)
C NAC feeding following exposure to P.e
w1118 iso
Female
Male
n= 121
n= 118
n= 118
n= 121
0.5h P.e - suc
0.5h P.e - NAC
Hours post-exposure
C'
log(HR) (95% CI) relative to P.e - suc
females
males
D Gut-specific silencing of Nox
Female
Male
n= 110
n= 98
n= 105
n= 84
mex>
attp2 (ctrl)
Nox RNAi
Hours post-exposure
D'
log(HR) (95% CI) relative to mex>attp2 RNAi
females
males
E Gut-specific silencing of Nox
n= 110
n= 119
n= 105
n= 122
mex>
attp2 (ctrl)
Nox RNAi
E'
F Gut-specific silencing of Duox
n= 110
n= 117
n= 105
n= 104
mex>
attp2 (ctrl)
Duox RNAi
F'
log(HR) (95% CI) relative to mex>attp40 RNAi
G Duox expression
bulk RNA seq
w1118 iso
FPKM
F suc
M suc
F 6h P.e
M 6h P.e
F 16h P.e
M 16h P.e
H Oxidative stress
Female
Relative DCF (%) / µg protein
ns
*
1h P.e
4h P.e
mex> attp2 (ctrl)
mex> Duox RNAi
I H2O2 levels
Female
log10(H2O2 (µmol/mg))
ns
ns
J Gut-specific overexpression of SOD1
n= 110
n= 112
n= 91
n= 114
mex>
GFP
SOD1
J'
log(HR) (95% CI) relative to mex>GFP
K Gut-specific overexpression of SOD1
n= 100
n= 100
n= 80
n= 79
myo1Ats>
mCherry RNAi
SOD1
K'
log(HR) (95% CI) relative to myo1Ats>mCherry RNAi
L Gut-specific overexpression of SOD2
n= 106
n= 87
n= 122
n= 105
myo1Ats>
mCherry RNAi
SOD2
L'
log(HR) (95% CI) relative to myo1Ats>mCherry RNAi
M Gut-specific overexpression of SOD1 - P.e+PQ
P.e
PQ
P.e+PQ
myo1Ats>mCherry RNAi females
myo1Ats>SOD1 females
n= 80
n= 78
n= 81
n= 100
n= 100
n= 98
M'
log(HR) (95% CI) relative to myo1Ats>mCherry RNAi P.e females
PQ
P.e+PQ
P.e
myo1Ats> mCherry RNAi
myo1Ats> SOD1

Fig. S6. Cytosolic but not mitochondrial oxidative stress determines female susceptibility to infection.

(A) H_2O_2 levels normalized to protein content (μmol/mg) of homogenized female and male *w^{1118}* iso gut samples. Data is shown $\log_{10}$-transformed values. (N = 3-4 independent samples, n = 30 guts per sample). Treatment: sucrose control and 1 h, 4 h post *P. entomophila* infection. Mean ± SE. Significance determined by two-way ANOVA with Tukey's multiple comparisons test (interaction: Sex × Treatment, p = ns (0.4270).

(B and B') Survival curves with 95% confidence intervals (shaded area) and hazard ratios with 95% confidence intervals of female and male *w^{1118}* iso flies upon exposure to paraquat (20mM paraquat, 2,5% sucrose in 4 h protocol).

(C and C') Survival curves with 95% confidence intervals (shaded area) and hazard ratios with 95% confidence intervals of female and male *w^{1118}* iso flies upon exposure to *P. entomophila* (4 h protocol) followed by sucrose (control) or N-Acetyl-L-Cystein (NAC) -supplemented sucrose.

(D and D') Survival curves with 95% confidence intervals (shaded area) and hazard ratios with 95% confidence intervals of female and male *mex>attp2* RNAi (control) and *mex>nox* RNAi (BL32433).

(E and E') Survival curves with 95% confidence intervals (shaded area) and hazard ratios with 95% confidence intervals of female and male *mex>attp2* RNAi (control) and *mex>nox* RNAi (BL32902).

(F and F') Survival curves with 95% confidence intervals (shaded area) and hazard ratios with 95% confidence intervals of female and male *mex>attp2* RNAi (control) and *mex>duox* RNAi (BL33975).

(G) Expression of *Duox* (FBgn0031464) measured by bulk RNA-seq in guts of female (F) and male (M) *w^{1118}* iso flies under sucrose-fed (control) conditions and at 6 h and 16 h post exposure to *P. entomophila*. Each dot represents a biological replicate (N =3). Mean ± SEM.

(H) Reactive oxygen species in female *mex>attp2* RNAi (control) and *mex>duox* RNAi (BL33975) measured as percent of 2′,7′-dichlorofluorescein (DCF) relative fluorescence units (RFU) normalized per protein of homogenized gut samples. (N = 4 independent samples represented by different shape, n = 30 guts per sample). Treatment: 1 h and 4 h post *P. entomophila* infection. Mean ± SE. Significance determined by two-way ANOVA with with Tukey's multiple comparisons test Interaction: Genotype × Treatment, p = ns (0.1591).

(I) H_2O_2 levels in female *mex>attp2 RNAi* (control) and *mex>duox RNAi* (BL33975) normalized to protein content (μmol/mg) of homogenized in female and male *w^{1118}* iso flies *w^{1118}* iso gut samples. Data is shown $\log_{10}$-transformed values. (N = 4 independent samples, n = 30 guts per sample). Treatment: 1 h and 4 h post *P. entomophila* infection. Mean ± SE. Significance determined by two-way ANOVA with Tukey's multiple comparisons test Interaction: Genotype × Treatment, p = ns (0.7566).

(J and J') Survival curves with 95% confidence intervals (shaded area) and hazard ratios with 95% confidence intervals of female and male *mex>UAS-GFP* (control) and *mex>UAS-SOD1* (BL24750).

(K and K') Survival curves with 95% confidence intervals (shaded area) and hazard ratios with 95% confidence intervals *myo1A^{ts}>mCherry* RNAi (control) and *myo1A^{ts}>UAS-SOD1* (BL24574) upon exposure to *P. entomophila.*

(L and L') Survival curves with 95% confidence intervals (shaded area) and hazard ratios with 95% confidence intervals *myo1A^{ts}>mCherry* RNAi (control) *myo1A^{ts}>UAS-SOD2* upon exposure to *P. entomophila.*

(M and M') Survival curves with 95% confidence intervals (shaded area) and hazard ratios with 95% confidence intervals of female *myo1A^{ts}>mCherry* RNAi (control) and *myo1A^{ts}>UAS-SOD1* (BL24750) flies upon exposure to *P. entomophila*, paraquat, and *P. entomophila*/paraquat mixture.

*p < 0.05, **p < 0.01, ***p < 0.001, ****p < 0.0001. For detailed sample sizes and statistical analyses, see Table S9.

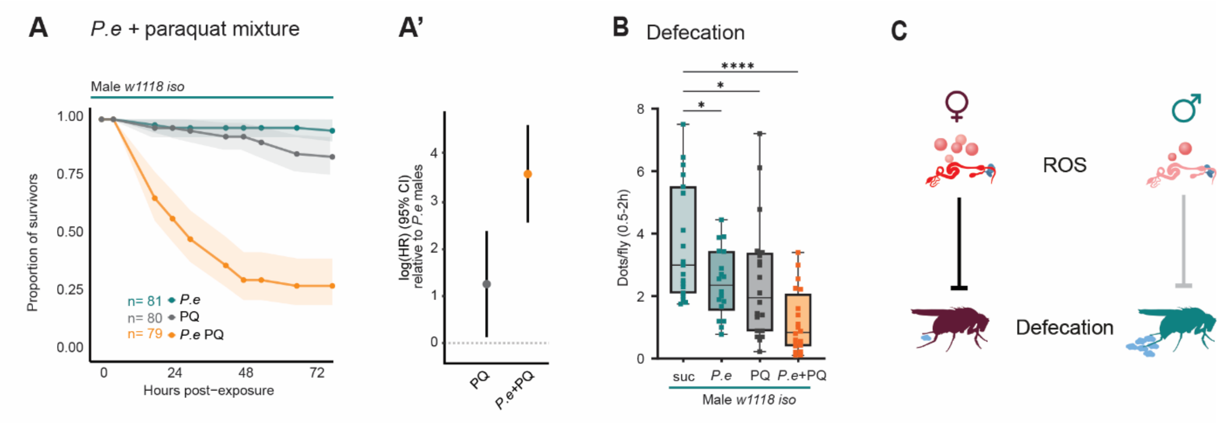

Fig. S7. Paraquat sensitizes males to P. entomophila infection by inhibiting defecation.

(A and A’) Survival curves with 95% confidence intervals (shaded area) and hazard ratios with 95% confidence intervals of male w^{1118} iso flies upon exposure to *P. entomophila*, paraquat, and *P. entomophila*/paraquat mixture.

(B) The defecation of male w^{1118} iso flies measured 0.5 to 2 h after exposure to blue-dyed sucrose (control), *P. entomophila*, paraquat, or *P. entomophila*/paraquat mixture in 0.5 h protocol. Significance by two-way ANOVA with Šídák's multiple comparisons test.

(C) Graphical summary illustrating the link between sexual dimorphism in intestinal infection-induced ROS levels and defecation.

For detailed sample sizes and statistical analyses, see Table S9.

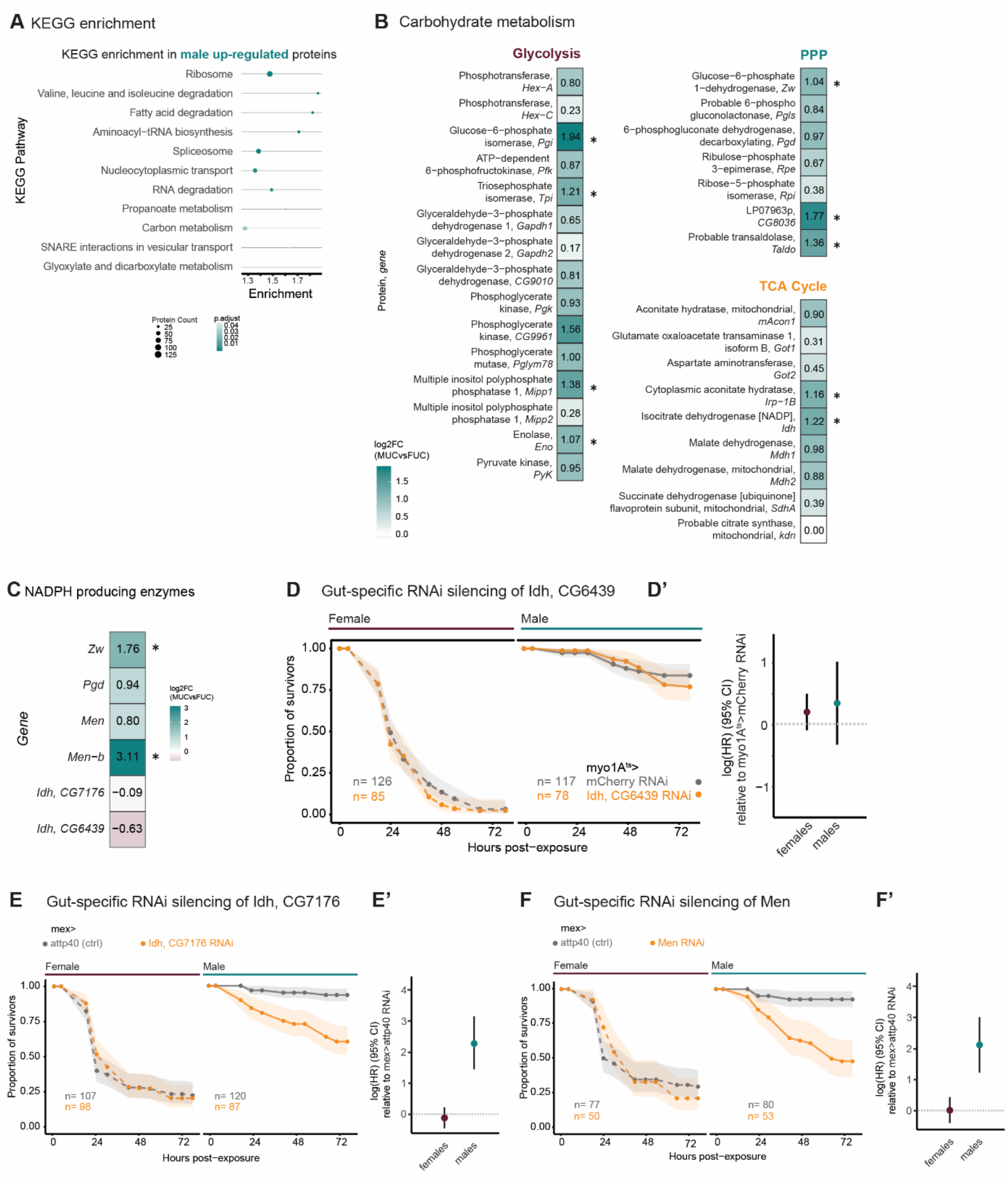

Fig. S8. Male bias in NADPH contributes to gut antioxidant capacity needed for survival.

(A) KEGG pathway analysis of proteins differentially-regulated between male and female guts under sucrose-fed (control) conditions.

(B) Heatmap showing abundance difference (log_2FC) of proteins involved in glycolysis, PPP, and TCA cycle between male and female guts under sucrose-fed (control) conditions. Significance (padj < 0.05, |log2FC| > 1) indicated by *.

(C) Heatmap showing expression level difference (log_2FC) of NADPH producing enzymes between male and female guts under sucrose-fed (control) conditions. Significance (padj < 0.1, |log2FC| > 1.5) indicated by *.

(D and D') Survival curves with 95% confidence intervals (shaded area) and hazard ratios with 95% confidence intervals of female and male *myo1A^{ts}>mCherry* RNAi (control) and *myo1A^{ts} >Idh, CG6439* RNAi upon exposure to *P. entomophila*.

(E – F') Survival curves with 95% confidence intervals (shaded area) and hazard ratios with 95% confidence intervals of female and male *mex>mCherry* RNAi (control) and: **(E and E')** *mex>Idh*, *CG7176* RNAi (flies with knockout of NADPH producing enzyme Idh in the enterocytes), **(F and F')** *mex>Men* RNAi (flies with knockout of NADPH producing enzyme Men in the enterocytes) upon exposure to *P. entomophila*.

For detailed sample sizes and statistical analyses, see Table S9.

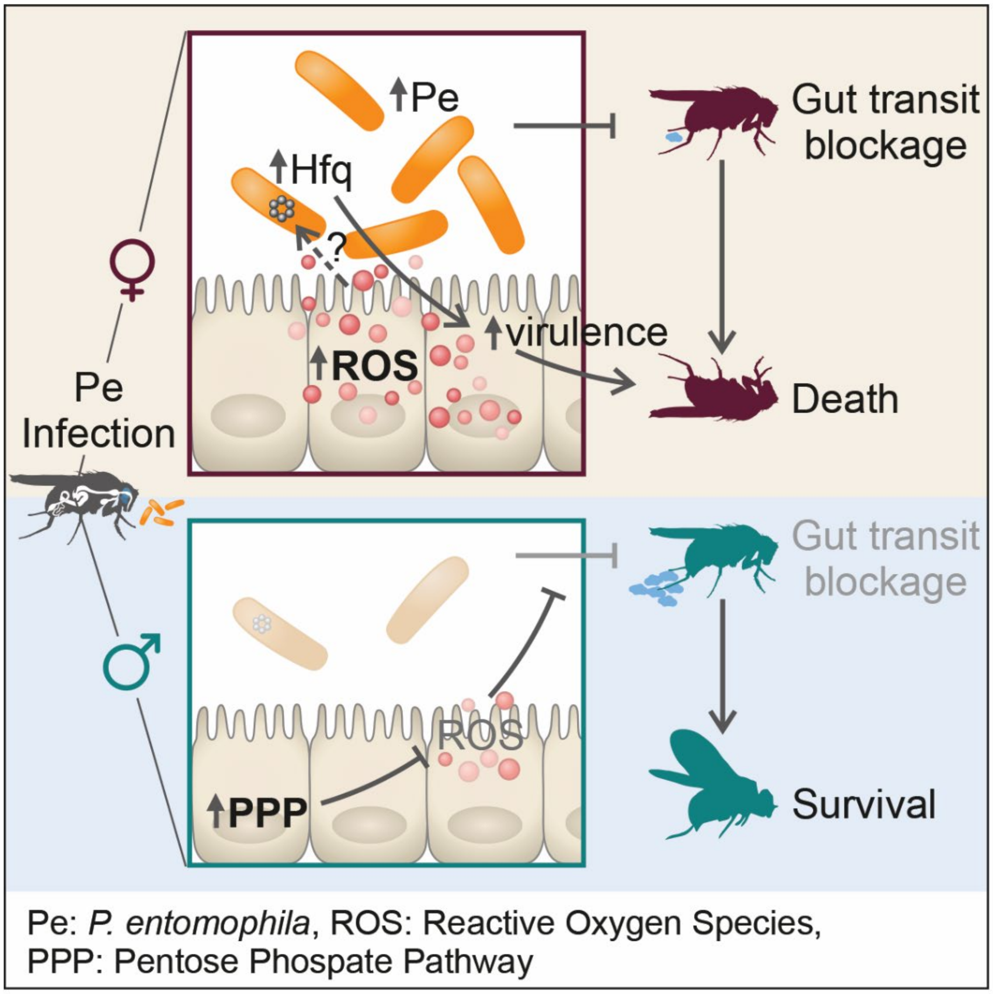

Fig. S9. Graphical summary illustrating the proposed model explaining the mechanism of sexual dimorphism in *Drosophila* susceptibility to *P. entomophila* gut infection.
See main text for details

Movies

Supplementary movie S1 (separate file). Representative movie of a female w^{1118} iso gut showing contractions 1 h after sucrose feeding, recorded in Schneider's medium. The video represents a 1 min period. Video duration: 30 s (2 frames per second).

Supplementary movie S2 (separate file). Representative movie of a female w^{1118} iso gut showing contractions 1 h after exposure to *P. entomophila*, recorded in Schneider's medium. The video represents a 1 min period. Video duration: 30 s (2 frames per second).

Supplementary movie S3 (separate file). Representative movie of a male w^{1118} iso gut showing contractions 1 h after sucrose feeding, recorded in Schneider's medium. The video represents a 1 min period. Video duration: 30 s (2 frames per second).

Supplementary movie S4 (separate file). Representative movie of a male w^{1118} iso gut showing contractions 1 h after exposure to *P. entomophila*, recorded in Schneider's medium. The video represents a 1 min period. Video duration: 30 s (2 frames per second).

Tables

Dataset S1 (separate file). Excel tables listing genes differentially-expressed under infection conditions.

Dataset S2 (separate file). Excel tables listing proteins differentially-expressed under infection conditions.

Dataset S3 (separate file). Excel tables listing GWAS-identified SNPs and candidate genes.

Dataset S4 (separate file). Excel tables listing transcripts and proteins differentially-expressed between male and female guts under basal conditions.

Dataset S5 (separate file). Excel tables listing *P. entomophila* proteins with differential abundance between male and female guts.

Dataset S6 (separate file). Excel tables with data used to generate graphs.

Dataset S7 (separate file). Excel table with the list of fly lines used in this study.

Dataset S8 (separate file). Excel table with the parameter settings of target metabolites measured in this study.

Dataset S9 (separate file). Excel tables reporting the details of statistical analysis.

SI References

1. J. C. Regan, *et al.*, Sex difference in pathology of the ageing gut mediates the greater response of female lifespan to dietary restriction. *eLife* **5** (2016).

2. Z. Zhai, X. Huang, Y. Yin, Beyond immunity: The Imd pathway as a coordinator of host defense, organismal physiology and behavior. *Developmental & Comparative Immunology* (2017). https://doi.org/10.1016/J.DCI.2017.11.008.

3. B. Hudry, *et al.*, Sex Differences in Intestinal Carbohydrate Metabolism Promote Food Intake and Sperm Maturation. *Cell* **178**, 901-918.e16 (2019).

4. N. P. du Sert, *et al.*, The ARRIVE guidelines 2.0: Updated guidelines for reporting animal research. *PLOS Biology* **18**, e3000410 (2020).

5. A. Arias-Rojas, D. Frahm, R. Hurwitz, V. Brinkmann, I. Iatsenko, Resistance to host antimicrobial peptides mediates resilience of gut commensals during infection and aging in Drosophila. *Proceedings of the National Academy of Sciences* **120**, e2305649120 (2023).

6. I. Iatsenko, J.-P. Boquete, B. Lemaitre, Microbiota-Derived Lactate Activates Production of Reactive Oxygen Species by the Intestinal NADPH Oxidase Nox and Shortens Drosophila Lifespan. *Immunity* **49**, 929-942.e5 (2018).

7. S. Chakrabarti, P. Liehl, N. Buchon, B. Lemaitre, Infection-induced host translational blockage inhibits immune responses and epithelial renewal in the Drosophila gut. *Cell Host Microbe* **12**, 60–70 (2012).

8. E. Bode, *et al.*, Promoter Activation in Δhfq Mutants as an Efficient Tool for Specialized Metabolite Production Enabling Direct Bioactivity Testing. *Angewandte Chemie International Edition* **58**, 18957–18963 (2019).

9. E. J. Du, *et al.*, TrpA1 Regulates Defecation of Food-Borne Pathogens under the Control of the Duox Pathway. *PLoS genetics* **12**, e1005773 (2016).

10. S. Anders, W. Huber, Differential expression analysis for sequence count data. *Genome Biology* **11**, R106 (2010).

11. T. Müller, *et al.*, Automated sample preparation with SP3 for low-input clinical proteomics. *Molecular Systems Biology* **16**, e9111 (2020).

12. V. Demichev, C. B. Messner, S. I. Vernardis, K. S. Lilley, M. Ralser, DIA-NN: neural networks and interference correction enable deep proteome coverage in high throughput. *Nat Methods* **17**, 41–44 (2020).

13. The UniProt Consortium, UniProt: the universal protein knowledgebase in 2021. *Nucleic Acids Research* **49**, D480–D489 (2021).

14. A. Gomes, E. Fernandes, J. L. F. C. Lima, Fluorescence probes used for detection of reactive oxygen species. *Journal of Biochemical and Biophysical Methods* **65**, 45–80 (2005).
